# Patient-associated mutations in *Drosophila* Alk perturb neuronal differentiation and promote survival

**DOI:** 10.1242/dmm.049591

**Published:** 2022-08-16

**Authors:** Kathrin Pfeifer, Georg Wolfstetter, Vimala Anthonydhason, Tafheem Masudi, Badrul Arefin, Mats Bemark, Patricia Mendoza-Garcia, Ruth H. Palmer

**Affiliations:** 1 Department of Medical Biochemistry and Cell Biology, Institute of Biomedicine, Sahlgrenska Academy, University of Gothenburg, SE-405 30 Gothenburg, Sweden; 2 Department of Microbiology and Immunology, Mucosal Immunobiology and Vaccine Center, Institute of Biomedicine, University of Gothenburg, SE-405 30 Gothenburg, Sweden

**Keywords:** Neuroblast, Tumor, Brain, Visceral mesoderm, Signaling, RTK, Neurogenesis, Neuroblastoma

## Abstract

Activating anaplastic lymphoma kinase (ALK) receptor tyrosine kinase (RTK) mutations occur in pediatric neuroblastoma and are associated with poor prognosis. To study ALK-activating mutations in a genetically controllable system, we employed CRIPSR/Cas9, incorporating orthologs of the human oncogenic mutations *ALK^F1174L^* and *ALK^Y1278S^* in the *Drosophila Alk* locus. *Alk^F1251L^* and *Alk^Y1355S^* mutant *Drosophila* exhibited enhanced Alk signaling phenotypes, but unexpectedly depended on the Jelly belly (Jeb) ligand for activation. Both *Alk^F1251L^* and *Alk^Y1355S^* mutant larval brains displayed hyperplasia, represented by increased numbers of Alk-positive neurons. Despite this hyperplasic phenotype, no brain tumors were observed in mutant animals. We showed that hyperplasia in *Alk* mutants was not caused by significantly increased rates of proliferation, but rather by decreased levels of apoptosis in the larval brain. Using single-cell RNA sequencing, we identified perturbations during temporal fate specification in *Alk^Y1355S^* mutant mushroom body lineages. These findings shed light on the role of Alk in neurodevelopmental processes and highlight the potential of Alk-activating mutations to perturb specification and promote survival in neuronal lineages.

This article has an associated First Person interview with the first author of the paper.

## Abstract (precis)

**Summary:** Orthologous neuroblastoma patient-derived point mutations in the *Drosophila* Anaplastic lymphoma kinase receptor tyrosine kinase drive aberrant signaling, resulting in decreased neuronal apoptosis and cell fate changes.

## INTRODUCTION

Neuroblastoma is the most common and deadly extracranial solid tumor in children (Maris, 2010; Tolbert and Matthay, 2018). Approximately 10% of neuroblastoma harbor mutations in the anaplastic lymphoma kinase (ALK) receptor tyrosine kinase (RTK), providing an important therapeutic target (Trigg and Turner, 2018). Failure of differentiation in the neural crest lineage during development is thought to result in aggressive neuroblastoma tumors with poor prognosis (Marshall et al., 2014; Tomolonis et al., 2018). Such tumors often harbor genetic aberrations such as *MYCN* amplification as well as amplification and/or gain-of-function mutations in *ALK* (Carén et al., 2008; Chen et al., 2008; George et al., 2008; Hallberg and Palmer, 2013; Janoueix-Lerosey et al., 2008; Mossé et al., 2008). Understanding the mechanisms underlying the contribution of ALK mutations to neuroblastoma development is important, and highly controlled genetic models to investigate their function *in vivo* provide critical insight.

ALK is mainly expressed in the central and peripheral nervous system (Iwahara et al., 1997; Vernersson et al., 2006), and *Alk* knockout mice are viable with mild phenotypes (Berry et al., 2012; Bilsland et al., 2008; Borenäs et al., 2021; Cazes et al., 2014; Ono et al., 2019; Orthofer et al., 2020; Witek et al., 2015). Knock-in mice harboring neuroblastoma-associated point mutations of *Alk* do not exhibit spontaneous tumor formation after birth but show neuronal hyperplasia (Berry et al., 2012; Borenäs et al., 2021; Cazes et al., 2014; Ono et al., 2019). In zebrafish and chicken models, Alk family RTKs are expressed in neural tissues and play a role during development of the neural crest (Fadeev et al., 2018; Mo et al., 2017; Vieceli and Bronner, 2018; Yao et al., 2013). However, the exact role of ALK in the nervous system and how it relates to development of neuroblastoma is poorly understood.

*Drosophila* Alk is indispensable for the embryonic development of the visceral muscles (Englund et al., 2003; Lee et al., 2003; Lorén et al., 2003; Stute et al., 2004). Alk signaling in response to the Jelly belly (Jeb) ligand engages the Ras–Raf–MAPK pathway, leading to specification of founder cell fate, which is critical for visceral muscle fusion (Englund et al., 2003; Lee et al., 2003; Lorén et al., 2003; Stute et al., 2004). During larval stages, Alk is robustly expressed in the central nervous system (CNS), where it performs multiple functions, including regulation of neuronal targeting and survival, synapse development and body size regulation, brain sparing, longevity, memory, circadian rhythm, ethanol response and learning (Bazigou et al., 2007; Cheng et al., 2011; Gouzi et al., 2018, 2011; Kumar et al., 2021; Lasek et al., 2011; Pecot et al., 2014; Rohrbough and Broadie, 2010; Rohrbough et al., 2013; Weiss et al., 2017, 2012; Woodling et al., 2020).

In the fruit fly, the process of neurogenesis, including the role of neuroblasts (NBs) and neuronal fate specification processes, is well-characterized, with each NB producing a distinct series of neurons (Yu et al., 2006). In the central brain, two types of NBs that are classified as Type I (NB I) and Type II (NB II) create distinct lineages (Doe, 2008; Knoblich, 2008; Urbach and Technau, 2004). Diversity of neurons in the *Drosophila* nervous system is achieved by highly regulated consecutive expression of transcription factors, which generate unique combinatorial transcription factor codes within NBs and their progeny (Liu et al., 2015; Syed et al., 2017a,b; Yang et al., 2016). The final complement of neurons is achieved through a binary, Notch-dependent manner via apoptosis, which occurs after division of the ganglion mother cell (GMC) (Pinto-Teixeira et al., 2016).

Alk has previously been described to function within the mushroom body (MB), where it regulates sleep behavior and long-term memory formation (Bai and Sehgal, 2015; Gouzi et al., 2011). The MB is a bilateral structure in the *Drosophila* brain that contains neurons that arise from four NBs per brain hemisphere, ultimately producing ∼2000 so-called Kenyon cells in the adult fly (Crittenden et al., 1998). It contains mainly three neuronal cell types: early-born γ-neurons, middle-born α′β′-neurons and late-born αβ-neurons, which form two dorsal lobes (α and α′) and three medial lobes (β, β′ and γ) (Crittenden et al., 1998; Ito et al., 1997; Lee et al., 1999). The γ-lobe undergoes remodeling during pupation in response to Ecdysone receptor B1 (EcR-B1; also referred to as EcR) activity (Lee et al., 2000), whereas the α′- and β′-lobe neurons are specified by expression of the Maternal gene required for meiosis (Mamo) BTB/POZ-containing C2H2-zinc finger transcription factor, which also maintains their cell fate (Liu et al., 2019).

Here, we exploited the genetically controlled *Drosophila* model system to investigate the effect of *Alk* mutation during neurogenesis. To do this, we generated two *Alk* alleles, *Alk^F1251L^* and *Alk^Y1355S^*, which are orthologous to human *ALK^F1174L^* and *ALK^Y1278S^* mutations in neuroblastoma patients. Remarkably, although *Alk^Y1355S^* exhibits ectopic Alk signaling in the embryonic visceral mesoderm (VM), this mutant receptor is still ligand dependent. Although brains of both *Alk^F1251L^* and *Alk^Y1355S^* mutants exhibited reduced apoptosis resulting in a mild hyperplasia, mutation of *Alk* alone was not sufficient to drive spontaneous tumor development in the *Drosophila* brain. Single-cell RNA-sequencing (scRNA-seq) analysis of *Alk^Y1355S^* larval brains led the identification of differentially expressed genes, including *mamo*. Because both Mamo and Alk have described functions in the MB (Gouzi et al., 2011; Liu et al., 2019; Rossi and Desplan, 2020), we focused on Mamo in the MB lineage. Aberrant *Alk^Y1355S^* signaling in the MB lineage leads to precocious Mamo expression in γ-neurons during wandering third-instar (wL3) larval stages, reflecting defects in neuronal fate specification in MB NB lineages that persist into adulthood. These results provide novel insight into the effect of oncogenic Alk mutations on neuronal specification and survival during development.

## RESULTS

### *Alk^F1251L^* and *Alk^Y1355S^* mutant alleles exhibit gain-of-function activity

**Fig. 1.**
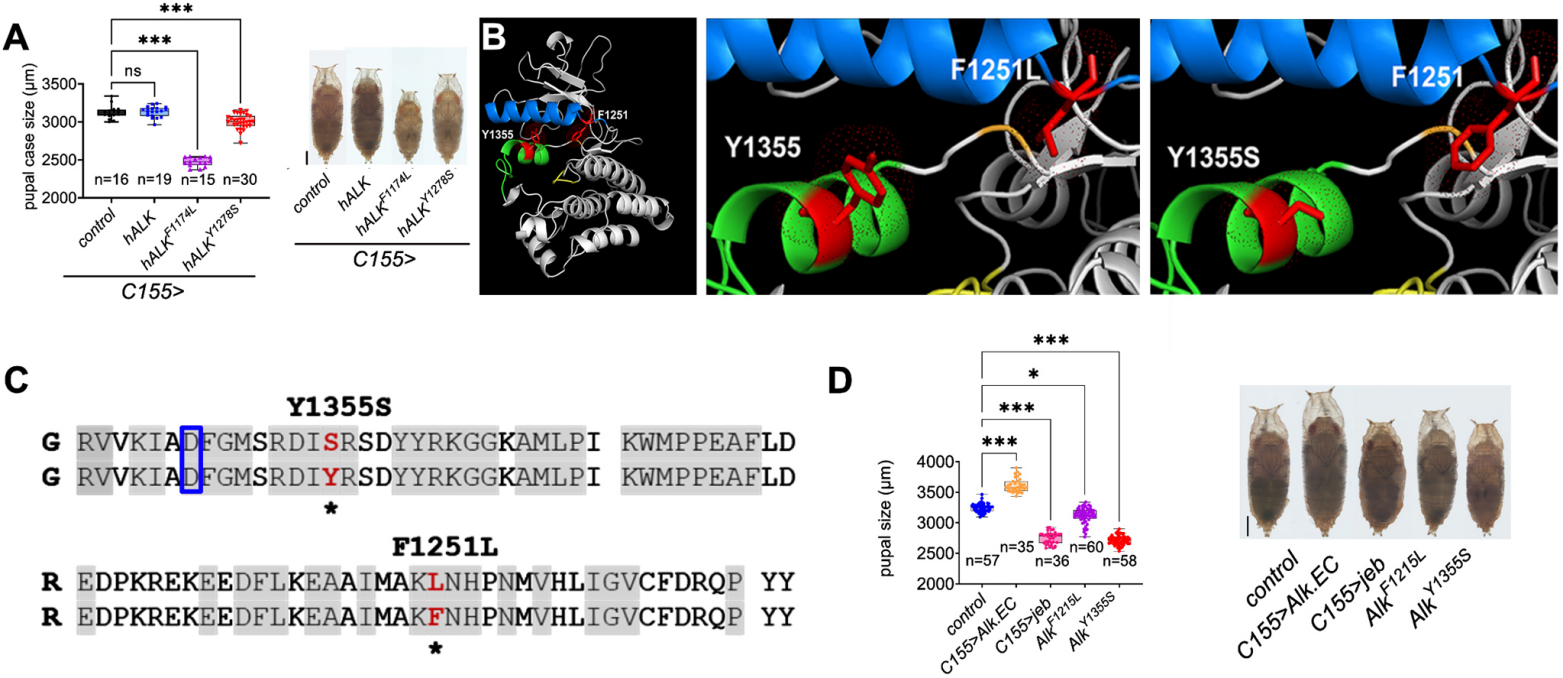
**CRISPR/Cas9-mediated generation of neuroblastoma-associated *Alk* mutations in *Drosophila*.** (A) Pupal size analysis of animals expressing human ALK transgenes pan-neuronally (*C155/+>hALK/+*, *C155/+>hALK^F1174L^*/+ or *C155/+>hALK^Y1278S^*/+). *n*=number of animals analyzed. One-way ANOVA, Dunnett's multiple comparison test; ***P<0.001. ns, non-significant. Scale bar: 500 µm. (B) Structural model of the *Drosophila* Alk kinase domain indicating residues F1251 and Y1355. Blue, alpha-C helix; green, activation loop; red, amino acids F1251 and Y1355. (C) Amino acid alignments between the wild-type Alk sequence (bottom line in Y1355S and F1251L) and *Alk^F1251L^* or *Alk^Y1355S^*, respectively. Asterisks mark amino acid changes. Boxed D indicates aspartic acid at residue 1347. (D) Pupal size analysis of homozygous *Alk^F1251L^* and *Alk^Y1355S^*, as well as *C155/+>jeb/+* and *C155/+>Alk.EC/+*, animals*.* One-way ANOVA, Kruskal–Wallis test; ***P<0.001 and *P<0.014. Scale bar: 500 µm. *n*=animals analyzed in three experimental replicates. In box plots, the box represents the 25-75th percentiles, and the line indicates the median; whiskers indicate minimum and maximum values. Representative examples are shown.

Overexpression of either Alk or Jeb ligand in the *Drosophila* CNS results in a reduced pupal size phenotype that provides a sensitive readout for Alk activity (Gouzi et al., 2011; Mendoza-García et al., 2017; Wolfstetter et al., 2017). To address whether the ability of oncogenic human ALK to drive the reduced pupal size phenotype is conserved, we employed the pan-neuronal *C155-Gal4* driver to overexpress either (1) wild-type human ALK, (2) a human ALK-F1174L gain-of-function mutant (representing an ALK hotspot mutation in neuroblastoma) or (3) an additional ALK-Y1278S gain-of-function neuroblastoma mutant (Umapathy et al., 2019; Vasseur et al., 2019). Expression of either ALK-F1174L or ALK-Y1278S mutant variants, but not wild-type ALK, resulted in a reduced pupal size phenotype, suggesting that ALK receptor signaling output is conserved between human and *Drosophila* (Fig. 1A). However, although informative, overexpression results in substantial modification of receptor numbers and signaling dynamics that can potentially lead to non-specific phenotypes. We therefore generated mutations in the endogenous *Alk* locus that model neuroblastoma patient mutations in a controlled genetic background, selecting two representative mutations – human *ALK-F1174L* and *ALK-Y1278S*. These gain-of-function ALK mutations are reported as constitutively active ALK mutations found in human neuroblastoma patients, with *ALK-F1174L* being a more frequently occurring ‘hot-spot’ mutation (Fig. 1B,C) (Umapathy et al., 2019; Vasseur et al., 2019). Sequence alignment analysis identified the equivalent residues in *Drosophila* Alk as *Alk-F1251* and *Alk-Y1355* (Fig. 1C; Fig. S1A). A CRISPR/Cas9-mediated homology-directed repair (HDR) strategy was used to generate *Alk^F1251L^* (phenylalanine to leucine at position 1251) and *Alk^Y1355S^* (tyrosine to serine at position 1355) mutations in the *Drosophila Alk* locus, allowing investigation of the effect of these patient-derived gain-of-function ALK mutations in the fly brain (Fig. S1B).

*Alk^F1251L^* and *Alk^Y1355S^* flies were homozygous viable and fertile. We performed two independent assays to characterize gain-of-function Alk activity: (1) analysis of pupal size and (2) analysis of Alk signaling in the embryonic VM. As previously reported, pan-neuronal expression of the Alk ligand Jeb (*C155>jeb*) resulted in a reduced pupal size phenotype (Fig. 1D), whereas inhibition of Alk signaling by overexpression of a dominant-negative Alk variant (*C155>Alk.EC*) increased pupal size (Fig. 1D). Homozygous *Alk^F1251L^* and *Alk^Y1355S^* animals displayed a reduced size phenotype, confirming them as Alk gain-of-function mutations (Fig. 1D). Notably, *Alk^Y1355S^* exhibited a stronger phenotype than *Alk^F1251L^*, indicating that these mutations are not equally strong gain-of-function mutations, and heterozygous (e.g. *Alk^Y1355S^/+*) pupae displayed a weaker phenotype than homozygous mutants, suggesting dosage sensitivity (Fig. S3A).

**Fig. 2.**
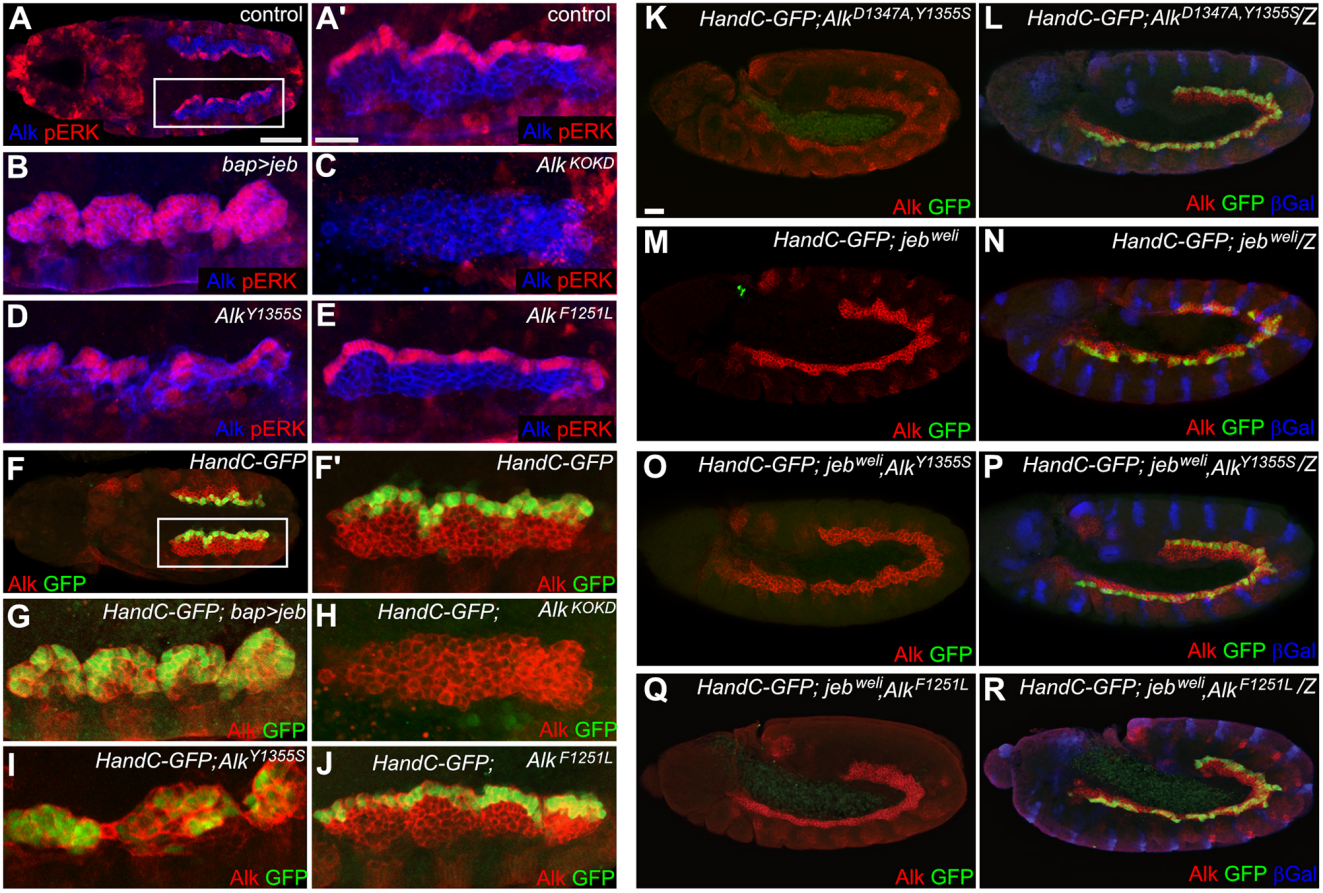
**Analysis of Alk signaling in the embryonic visceral mesoderm (VM) of *Alk^F1251L^* and *Alk^Y1355S^* mutants.** (A,A′) Phospho-ERK (pERK) staining in the VM founder cell (FC) row of control embryos. (A′) Magnification of boxed region in A. Scale bars: 50 µm (A) and 10 µm (A′). (B) Ectopic expression of Jeb with *bap-Gal4* (*bap/+>jeb/+*) leads to ERK phosphorylation within the whole VM. (C) No pERK staining is observed in *Alk^KO^* loss-of-function homozygous mutants. (D) Homozygous *Alk^Y1355S^* mutant embryos (21%) display ectopic pERK signals at stage 10/11 in VM cells adjacent to the FC row. *n*=155 embryos analyzed. (E) FC-specific pERK staining in homozygous *Alk^F1251L^* embryos. (F,F′) Control embryos display FC-specific expression of the *HandC-GFP* reporter in the VM. (F′) Magnification of F. (G) Ectopic expression of Jeb with *bap-Gal4* (*bap/+>jeb/+*) results in *HandC-GFP* expression in all VM cells. (H) *HandC-GFP* expression is absent in the VM of homozygous *Alk^KO^* embryos. (I) Ectopic HandC-GFP reporter expression was observed in *HandC-GFP*, *Alk^Y1355S^* embryos. (J) *HandC-GFP* expression in *Alk^F1251L^* homozygous embryos is similar to controls. (K) *Alk^Y1355S, D1347A^* homozygous double mutants do not show *HandC-GFP* reporter expression in the VM. Scale bar: 20 µm. (L) *HandC-GFP* expression in heterozygous *Alk^Y1355S, D1347A^*/*CyO, wg-lacZ* embryos. (M,N) *HandC-GFP* reporter expression in *jeb^weli^* homozygous mutants (M) and *jeb^weli^*/*CyO, wg-lacZ* heterozygous controls (N). (O,Q) *HandC-GFP* expression in *Alk^Y1355S^*, *jeb^weli^* (O) and *Alk^F1251L^, jeb^weli^* (Q) homozygous mutants. (P,R) *Alk^Y1355S^*, *jeb^weli^* (P) and *Alk^F1251L^, jeb^weli^* (R) heterozygous controls at stage 11. >150 embryos were analyzed. All embryos shown are in stage 10/11.

To further analyze Alk signaling in *Alk^F1251L^* and *Alk^Y1355S^* mutants, we turned to the embryonic VM (controls in Fig. 2A,A′,F,F′) using phospho-ERK (pERK) staining or *HandC-GFP* reporter expression as read-out (Englund et al., 2003; Lee et al., 2003; Lorén et al., 2003; Stute et al., 2004). In contrast to the restricted founder cell activation observed in controls (Fig. 2A,A′,F,F′), all Alk-expressing cells in the VM responded with robust pERK staining and *HandC-GFP* reporter activation (Fig. 2B,G) when Jeb was ectopically expressed (*bap>jeb*). In contrast, no detectable pERK staining or *HandC-GFP* reporter expression was observed in the *Alk^KO^* kinase domain deletion mutant (Wolfstetter et al., 2017) (Fig. 2C,H). Ectopic Alk signaling was also observed in 21% of the homozygous *Alk^Y1355S^* embryos (Fig. 2D,I; Fig. S2A,B). Surprisingly, no difference was seen between Alk signaling output in the VM of *Alk^F1251L^* mutants and control embryos (Fig. 2E,J). Taken together, these findings suggest that the *Alk^F1251L^* and *Alk^Y1355S^* alleles generated here represent *Drosophila Alk* gain-of-function alleles of differing strength, with *Alk^Y1355S^* representing a stronger gain-of-function allele than *Alk^F1251L^*.

### *Alk^Y1355S^* and *Alk^F1251L^* mutants are ligand dependent

To ensure that the phenotypes observed were specific for modification of the *Alk* locus, we combined a kinase-dead mutation (*Alk^D1347A^*) with the *Alk^Y1355S^* allele using CRISPR/Cas9-mediated HDR. *Alk^D1347A^* harbors a modification of the highly conserved DFG motif in the kinase domain, in which the ATP-γ-phosphate-binding aspartic acid 1347 is changed to alanine (Fig. 1C; Fig. S1A). As expected, homozygous *Alk^D1347A^* animals were lethal and failed to specify VM founder cells (Fig. S3B). Analysis of the *Alk^D1347A, Y1355S^* double mutant allele showed that the embryonic VM phenotype of *Alk^Y1355S^* was abrogated by the D1347A kinase-dead mutation, resulting in an *Alk* loss-of-function phenotype (Fig. 2K,L). Further, the reduced size phenotype of *Alk^Y1355S^/+* was rescued in *Alk^D1347A, Y1355S^/+* animals (Fig. S3A). Taken together, these data confirm that Alk kinase activity is required for signaling output in both wild-type and gain-of-function backgrounds, and that the phenotypes observed in the *Alk^Y1355S^* mutant allele are specific effects of increased Alk signaling and not due to CRISPR/Cas9 off-target effects.

We next investigated whether the *Alk^F1251L^* and *Alk^Y1355S^* alleles were ligand independent by testing their ability to rescue *jeb* loss-of-function mutants, which also fail to specify VM founder cells (Englund et al., 2003; Lee et al., 2003; Stute et al., 2004; Weiss et al., 2001) (Fig. 2M compared to control Fig. 2N). Remarkably, both *Alk^Y1355S^, jeb^weli^* and *Alk^F1251L^, jeb^weli^* double mutants were lethal and exhibited the *jeb^weli^* phenotype (Fig. 2O-R). These data clearly show that although *Alk^Y1355S^* and *Alk^F1251L^* exhibit a gain-of-function Alk signaling pupal size phenotype, they remain ligand dependent in the context of embryonic VM founder cell specification.

### Alk is expressed in mature neurons of the *Drosophila* larval brain

**Fig. 3.**
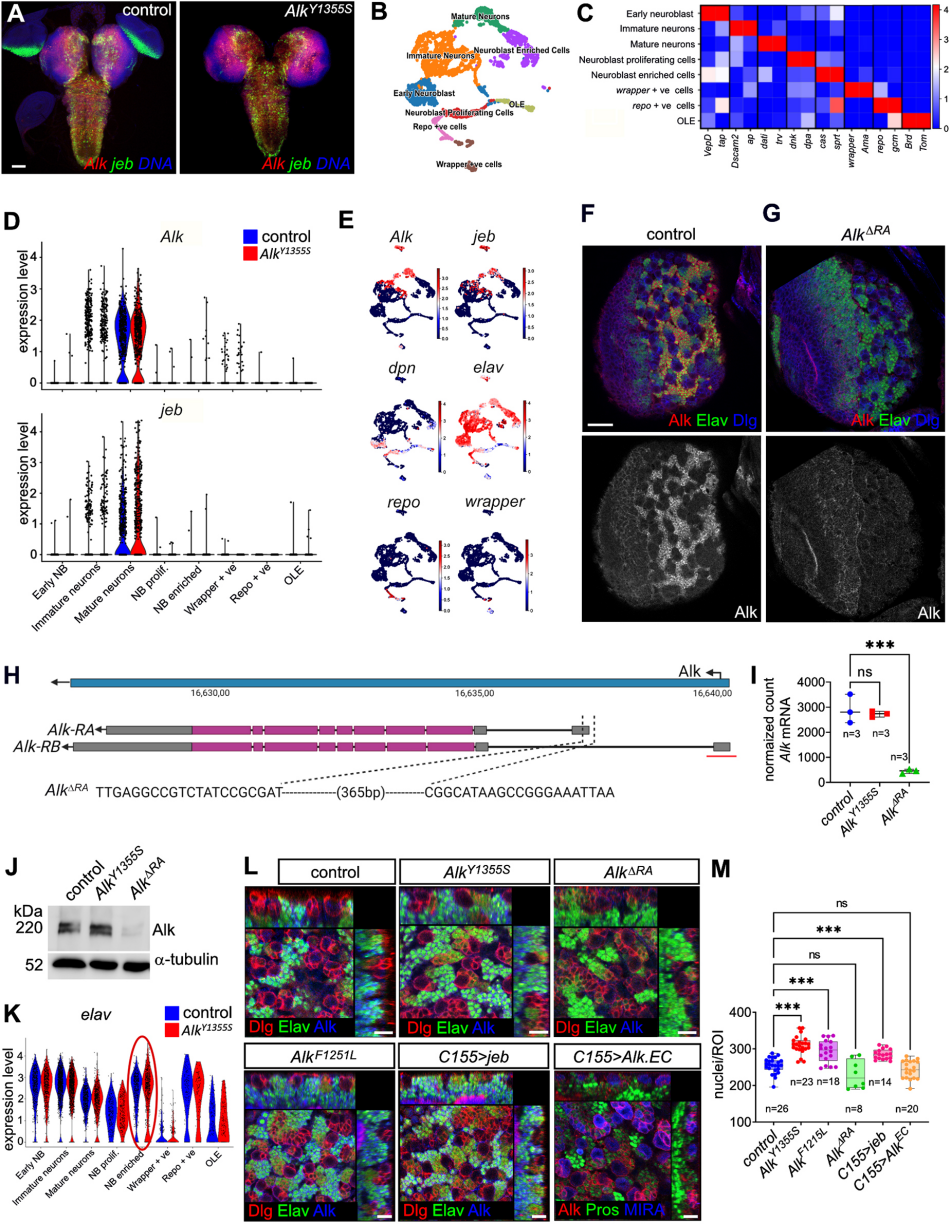
***Alk^F1251L^* and *Alk^Y1355S^* larval brains display hyperplasia.** (A) Expression of *Alk* (red) and *jeb* (green) mRNAs *in situ* are shown; DAPI is in blue. *n*>20. Two independent replicates. Scale bar: 50 µm. (B) Uniform manifold approximation and projection (UMAP) displaying the cellular heterogeneity of the control single-cell RNA-sequencing (scRNA-seq) dataset as eight cell types [early neuroblast (NB), NB-enriched cells, immature neurons, mature neurons, NB proliferating cells, optic lobe epithelium (OLE), Repo-positive cells and Wrapper-positive cells]. (C) Matrix plot visualizing canonical markers (two per cluster) defining the cellular heterogeneity of the scRNA-seq dataset. Scale indicates mean expression values. (D) Violin plots showing the mRNA expression of *Alk* and *jeb* across larval brain scRNA-seq clusters (blue denotes control and red denotes *Alk^Y1355S^*). (E) Feature plots visualizing mRNA expression of *Alk*, *jeb*, *elav*, *dpn*, *repo* and *wrapper* within the single-cell population. Color scale indicates expression level. (F,G) Alk protein expression in the central brain area [one wandering third-instar (wL3) larval brain hemisphere is shown], in control (F) and *Alk^ΔRA^* mutant (G). Alk (red), Elav (green) and Dlg (blue) are shown. Scale bar: 20 µm. *n*>30 larval brains in three replicates. (H) Schematic outlining the genomic organization of the *Alk* locus (blue). Intron–exon structure of both *Alk-RA* and *Alk-RB* transcripts is shown below. Open reading frame is in pink. The sequence of the *Alk^ΔRA^* mutant is shown below, and the region deleted in the previously described *Alk^ΔRB^* mutant (Mendoza-Garcia et al., 2017) is indicated for reference (red line). Schematic created with biorender.com. (I) *Alk* mRNA expression levels in control, homozygous *Alk^Y1355S^* and *Alk^ΔRA^* third-instar larval brain bulk RNA-seq. Three biological replicates (45 brains per sample) per genotype are shown. Ordinary one-way ANOVA, Dunnett's multiple comparison test; ***P<0.001. (J) Immunoblot indicating Alk protein levels in control, *Alk^Y1355S^* and *Alk^ΔRA^* third-instar larval brain lysates. Tubulin was employed as loading control. One representative image of two experimental replicates is shown. (K) Violin plot indicating *elav* mRNA expression across third-instar larval brain scRNA-seq clusters. Red oval highlights expression of *elav* in the NB-enriched cluster in *Alk^Y1355S^*. (L) Orthogonal projections of the wL3 central brain area showing Elav-positive perikarya at the NB level. Scale bars: 10 µm. (M) Nuclei count per region of interest (ROI) in homozygous *Alk^Y1355S^*, homozygous *Alk*^*F1251L*^, *C155/+>jeb/+*, homozygous *Alk^ΔRA^* and *C155/+>Alk^EC^/+*. In box plots, the box represents the 25-75th percentiles, and the line indicates the median; whiskers indicate minimum and maximum values. *n*=number of brain lobes analyzed in three independent replicates. One-way ANOVA, Dunnett's multiple comparison test; ***P<0.001. scRNA-seq data from 45 dissected and pooled wL3 larval brains were used for B-E and K. ns, not significant.

Previous studies have reported broad expression of *Alk* mRNA and protein in the *Drosophila* nervous system (Bazigou et al., 2007; Cheng et al., 2011; Gouzi et al., 2011; Lorén et al., 2003, 2001). We used fluorescent *in situ* hybridization chain reaction (HCR) to detect *Alk* and *jeb* mRNA in the CNS of control and *Alk^Y1355S^* animals. Both *Alk* and *jeb* mRNA were robustly expressed at similar levels in both control and *Alk^Y1355S^* third-instar (L3) larval brains (Fig. 3A). To further investigate the effect of the *Alk^Y1355S^* mutation, we analyzed L3 larval brains of control and *Alk^Y1355S^* alleles using 3′ scRNA-seq based on the 10x Genomics platform. In total, high-quality scRNA-seq data were collected for 3967 control and 4099 *Alk^Y1355S^* CNS cells and further analyzed with R (Seurat) and Python-based (Scanpy) pipelines. This analysis initially defined 19 cell clusters (Fig. S4A), which were merged based on canonical markers (Fig. S4B,D) into eight distinct clusters displayed on a uniform manifold approximation and projection (UMAP)-based two-dimensional projection (Fig. 3B). These eight clusters were defined as: early NBs, NB-enriched cells, immature neurons, mature neurons, NB proliferating cells, optic lobe epithelium (OLE), Repo-positive cells and Wrapper-positive cells. Canonical markers for these subsets were expressed in these clusters (Fig. 3C; Fig. S4D), and we identified several other unique marker genes for each subtype (Ariss et al., 2020; Brunet Avalos et al., 2019; Cattenoz et al., 2016; Estacio-Gómez et al., 2020; Michki et al., 2021) (Fig. S4E). In addition to these clusters, we identified two additional clusters representing hemocytes (cluster 3 in Fig. S4A) and cuticle-associated cells (cluster 18 in Fig. S4A,C) (Cattenoz et al., 2020; Dong et al., 2020; Evans et al., 2014; Öztürk-Çolak et al., 2016).

*Alk* and *jeb* were robustly expressed in the scRNA-seq dataset of both control and *Alk^Y1355S^* larval brains. Both *Alk* and *jeb* were predominantly expressed in mature neuronal lineages (Fig. 3D,E), including cholinergic, glutamatergic and GABAergic neurons (Fig. S4F). This is in keeping with previously described expression of Alk in the medulla of the optic lobe (Bazigou et al., 2007) and of Jeb in cholinergic neurons (Okamoto and Nishimura, 2015). Interestingly, we detected very little *Alk* or *jeb* at the mRNA level in either glial or NB-enriched cell populations (Fig. 3D,E).

We next investigated Alk protein expression in the L3 larval brain, confirming that Alk was strongly expressed in Elav-positive progeny of both Type I and Type II NBs (Fig. 3F). To examine the role of Alk further, we generated an *Alk* mutant allele (*Alk^ΔRA^*), specifically removing *Alk* expression in the larval brain (Fig. 3G,H). In previous work, transcripts of the *Alk-RB* isoform were specifically detected in the *Drosophila* embryonic VM (Mendoza-García et al., 2017). Employing isoform-specific probes for *Alk-RA* and *Alk-RB*, we confirmed the previously described VM-expression of *Alk-RB* (Mendoza-García et al., 2017) and revealed that the *Alk-RA* isoform is specifically expressed in the developing nervous system (Fig. S5A,B). Based on this, we employed CRISPR/Cas9-mediated targeted deletion to remove the *Alk-RA* transcript generating the *Alk^ΔRA^* mutant allele (Fig. 3H). Indeed, expression of Alk protein in the L3 larval central brain was significantly reduced, which was further confirmed by RNA-sequencing (RNA-seq) analysis (Fig. 3G,I) and immunoblotting (Fig. 3J). *Alk^ΔRA^* mutants were viable with no obvious defects during development, providing an excellent tool with which to further investigate Alk function in the larval brain.

### *Alk^F1251L^* and *Alk^Y1355S^* exhibit neuronal hyperplasia at larval stages

Because our scRNA-seq and Alk antibody staining highlighted robust expression of Alk in central brain neurons, we focused on this area for further investigation. A closer examination of the central brain area of *Alk^Y1355S^* and *Alk^F1251L^* identified increased numbers of neurons relative to controls (Fig. 3L). This increase in Elav-positive neurons was also observed on pan-neuronal expression of Jeb with *C155-Gal4* (Fig. 3L). Loss of Alk activity, either by overexpression of dominant-negative Alk (*C155>Alk.EC*) or in the *Alk^ΔRA^* mutant (Fig. 3L), did not significantly affect the numbers of Elav-positive neurons. These results were further strengthened by a slight increase in Elav-expressing cells in the *Alk^Y1355S^* scRNA-seq dataset (Fig. 3K). To quantify this phenotype, L3 larval brains were stained with 4′,6-diamidino-2-phenylindole (DAPI), and perikarya within a region of interest (ROI) at the NB level were counted. Significantly increased nuclei numbers were observed in both *Alk^Y1355S^* and *Alk^F1251L^* mutant alleles as well as on Jeb overexpression (*C155>Jeb*) (Fig. 3M). Notably, these observations are in keeping with observations of hyperplasia in the nervous system of *Alk* gain-of-function mouse models (Borenäs et al., 2021; Cazes et al., 2014). No significant change in neuron number was observed in L3 larval brains lacking Alk activity, either employing an Alk dominant-negative transgene (*C155>Alk.EC*) or the *Alk^ΔRA^* mutant allele, suggesting that Alk function is not critically required for differentiation. Thus, our data suggest that the larval brains of *Alk* gain-of-function alleles contain increased numbers of mature neurons.

### scRNA-seq analysis identifies perturbed neuronal identity in *Alk^Y1355S^* larval brains

**Fig. 4.**
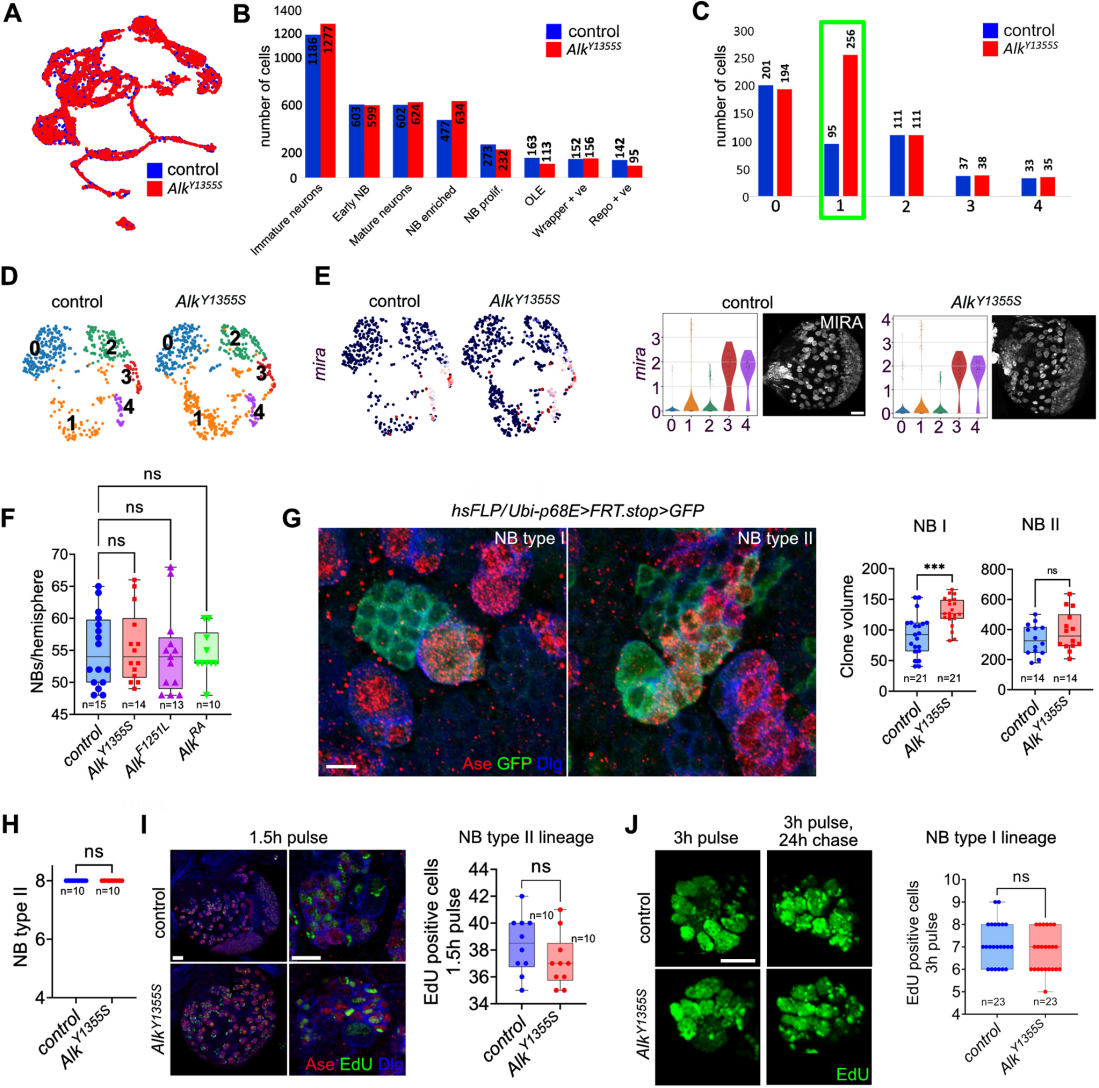
**Single-cell analysis identifies perturbed NB lineages in *Alk^Y1355S^* mutants.** (A) UMAP of integrated scRNA-seq control (blue) and *Alk^Y1355S^* (red) datasets. (B) Bar chart indicating the number of cells in each cluster across the whole brain cell population in both control (blue) and *Alk^Y1355S^* (red) scRNA-seq datasets. (C) Bar chart displaying cell distribution in a re-analysis of the NB-enriched cell population in B. Increased numbers of cells are observed in cluster 1 (green rectangle) of *Alk^Y1355S^* (red), compared to control (blue). (D) UMAPs showing the five sub-clusters (shown in C) identified in control and *Alk^Y1355S^* from the NB-enriched population. (E) UMAPs indicating *miranda* (*mira*) mRNA expression in both control and *Alk^Y1355S^* scRNA-seq datasets. Violin plots show *mira* mRNA expression and distribution in the subclusters of the NB-enriched cell population of control and *Alk^Y1355S^*, together with Mira expression in wL3 larval brains. Scale bar: 20 µm. (F) Quantification of NBs per brain hemisphere in homozygous *Alk^Y1355S^*, *Alk^F1251L^* and *Alk^ΔRA^* compared to controls. One-way ANOVA, Dunnett's multiple comparisons test. *n*=brain lobes analyzed. (G) GFP-positive clones derived from Type I and Type II NBs in either control or homozygous *Alk^Y1355S^* mutant backgrounds. Scale bar: 5 µm. Quantification shown on the right. Unpaired two-tailed Student's *t*-test; ***P<0.001. (H) Total number of Type II NBs per brain hemisphere in control and homozygous *Alk^Y1355S^*. Unpaired two-tailed Student's *t*-test, Kolmogorov–Smirnov test. *n*=brain lobes analyzed. (I) Quantification of EdU-positive cells in Type II NB lineages as identified by anti-Asense staining for control and homozygous *Alk^Y1355S^* wL3 larval brains. Unpaired two-tailed Student's *t*-test. Scale bars: 20 µm (left) and 5 µm (right). *n*=brain lobes analyzed. (J) EdU pulse-chase experiments show no significant difference in EdU-positive cells arising from NB Type I lineages in homozygous *Alk^Y1355S^* larval brains compared with control. EdU incorporation was quantified for 3 h pulse and 3 h pulse plus 24 h chase conditions. Unpaired two-tailed Student's *t*-test, Mann–Whitney test. Scale bar: 5 µm. *n*=brain lobes analyzed. scRNA-seq data from 45 dissected and pooled wL3 larval brains were used for A-E. In box plots, the box represents the 25-75th percentiles, and the line indicates the median; whiskers indicate minimum and maximum values. Representative examples are shown. ns, not significant.

Integration of *Alk^Y1355S^* and control scRNA-seq datasets was performed to identify shared and distinct cell identities (Fig. 4A), highlighting increased numbers of cells in the ‘NB-enriched’ cluster (Fig. 4B), and further suggesting that increased Alk activation may result in a perturbation of NB-derived lineages and populations. We therefore further interrogated this cell cluster in our scRNA-seq datasets. Re-analysis of the NB-enriched cell cluster resulted in five cell clusters (Fig. 4C,D). Although cluster 1 was enriched in *Alk^Y1355S^* compared to the control scRNA-seq dataset, similar numbers of *miranda* (*mira*)-positive NBs (clusters 3 and 4) were observed, and antibodies to Mira confirmed similar numbers of Mira-positive NBs in *Alk^Y1355S^* and control larval brains (Fig. 4E). We also counted NBs per brain hemisphere in *Alk^Y1355S^*, *Alk^F1251L^* and *Alk^ΔRA^*, but were unable to observe any change in overall NB number in *Alk* mutant brains, indicating that the additional neurons observed in *Alk^Y1355S^* and *Alk^F1251L^* brains were not a result of elevated NB numbers (Fig. 4F).

To address the dynamics of NB lineages, GFP-marked clones were generated in both control and *Alk^Y1355S^* backgrounds (Fig. 4G). Analysis of clones descended from Type I NBs revealed significant increase in volume in *Alk^Y1355S^* (Fig. 4G), in agreement with our earlier observation of increased numbers of Elav-positive perikarya in *Alk^Y1355S^* larval brains. Analysis of Type II NB clone size showed a non-significant trend towards increased volume (Fig. 4G), which was also reflected by similar numbers of Type II NBs per brain lobe (Fig. 4H). We further employed 5-ethynyl-2′-deoxyuridine (EdU) pulse-chase analysis, subjecting *Alk^Y1355S^* and control larvae to either (1) EdU pulse (1.5 h for NB II and 3 h for NB I) followed by immediate dissection or (2) EdU pulse followed by 24 h chase. EdU pulse-chase analyses did not reveal any significant differences in proliferation of Type I or Type II NBs when comparing *Alk^Y1355S^* larval brains with controls (Fig. 4I,J), indicating that the *Alk^Y1355S^* mutation does not increase proliferation rates in larval brains. Moreover, we employed the fluorescence ubiquitination cell cycle indicator (FUCCI) system in both wL3 larvae after eclosion and wL3 larvae, which enabled us to identify cells in different phases of the cell cycle (Zielke et al., 2014) (Figs S6 and S7). No differences in cell cycle phases were detected in *Alk^Y1355S^* compared to the control, indicating that proliferation is not enhanced at the examined time points. Thus, our scRNA-seq and subsequent experimental validations suggest that the hyperplasia observed in *Alk^Y1355S^* larval brains is not due to increased NB numbers or proliferation, implying that other mechanisms are responsible.

### NB quiescence proceeds as normal in *Alk^Y1355S^* mutants

**Fig. 5.**
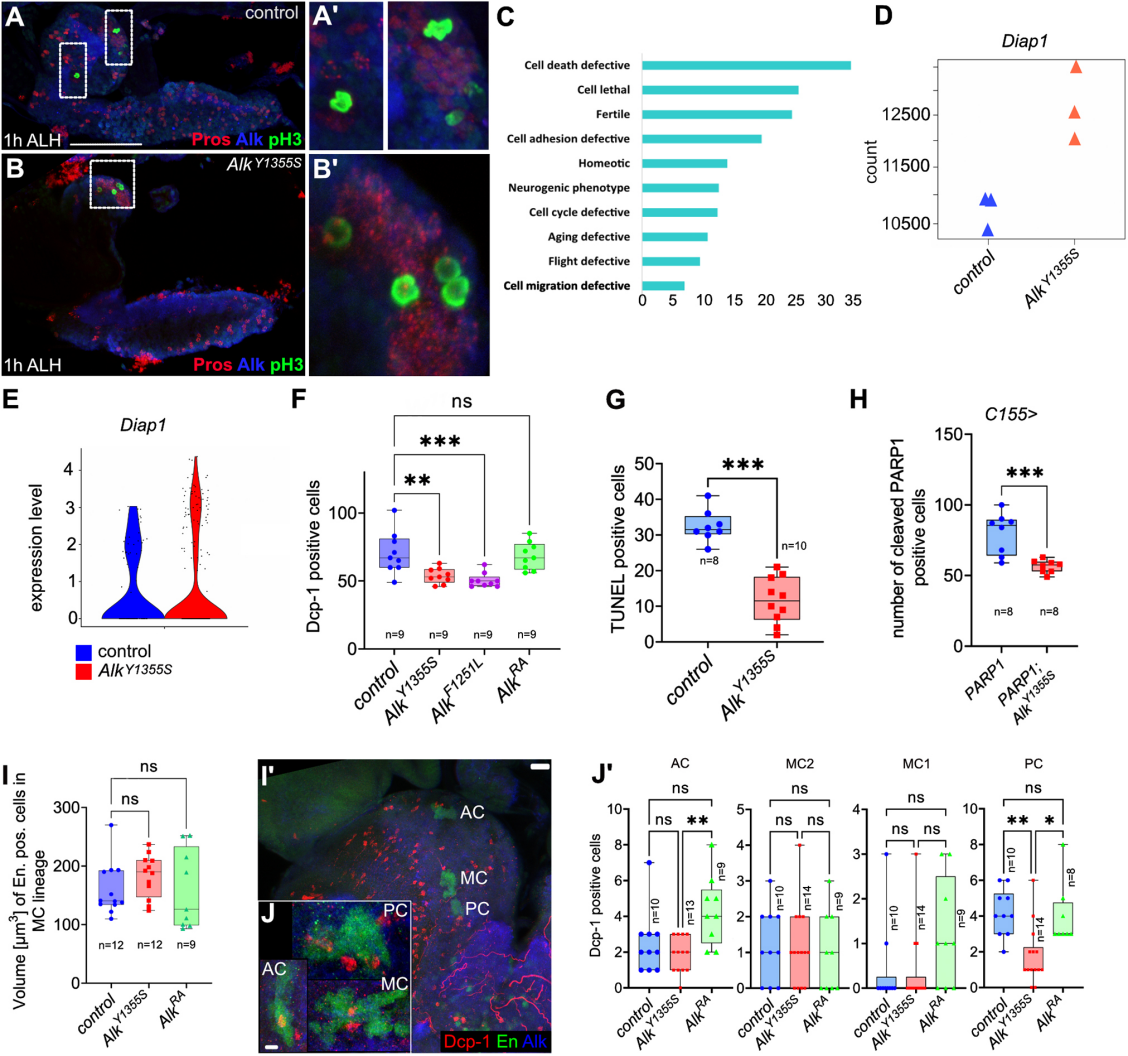
***Alk^Y1355S^* larval brains exhibit decreased levels of apoptosis.** (A,B) Four pH3-positive MB NBs are detected in both homozygous control (A) and *Alk^Y1355S^* (B) first-instar larval brains. Scale bar: 20 µm. *n*>5 brains. (C) Gene Ontology (GO) analysis of genes enriched in sub-cluster 1 of the reanalyzed NB-enriched cells cluster in *Alk^Y1355S^*, highlighting an enrichment in cell death-defective components in the *Alk^Y1355S^* scRNA-seq dataset. (D) Expression of *Diap1* in bulk RNA-seq from both control and homozygous *Alk^Y1355S^* larval brains. Three biological replicates (45 brains per sample) per genotype are shown. (E) Violin plot indicating *Diap1* expression in the NB-enriched cell population in homozygous *Alk^Y1355S^* and controls. (F) Quantification of Cleaved caspase-1 staining (Dcp-1) in the central brain area of homozygous *Alk^Y1355S^*, *Alk^F1251L^* and *Alk^ΔRA^* compared to controls. One-way ANOVA, Dunnett's multiple comparison test; ***P<0.001 and **P<0.005. *n*=number of brain lobes analyzed. (G) TUNEL analysis visualizing apoptotic cells in homozygous *Alk^Y1355S^* and controls. Unpaired two-tailed Student's *t*-test; ***P<0.001. *n*=number of brain lobes analyzed. (H) PARP1 reporter assay in control and homozygous *Alk^Y1355S^* larval brains. Unpaired two-tailed Student's *t*-test; ***P<0.001. (I,I′) Volume analysis of the Engrailed-positive MC sub-lineage in homozygous *Alk^Y1355S^*, *Alk^ΔRA^* and controls. Scale bar: 20 µm. One-way ANOVA, Kruskal–Wallis test. *n*=number of analyzed brain lobes. AC, anterior cluster; MC, medial cluster; PC, posterior cluster. (J,J′) Analysis of Dcp-1-positive neurons adjacent to the Engrailed sub-lineages AC, PC and MC in homozygous *Alk^Y1355S^*, *Alk^ΔRA^* and controls. Scale bar: 5 µm. One-way ANOVA, Kruskal–Wallis test. AC, **P<0.007; MC1, *P*>0.05; MC2, *P*>0.05; PC, *P<0.05, **P<0.005. *n*=number of brain lobes analyzed. scRNA-seq data from 45 dissected and pooled wL3 larval brains were used for C-E. In box plots, the box represents the 25-75th percentiles, and the line indicates the median; whiskers indicate minimum and maximum values. Representative examples are shown. ns, not significant.

In *Drosophila*, NBs enter a quiescent state during late embryogenesis and remain quiescent until larvae begin feeding (∼4 h after larval hatching) (Curt et al., 2019). Exceptions are the four MB NBs that do not undergo quiescence and continue dividing during late embryo and L1 stages (Ito and Hotta, 1992). To better understand the cause of the observed hyperplasia in *Alk^Y1355S^* larval brains, we therefore examined NB quiescence in these mutants. Newly hatched *Alk^Y1355S^* and control L1 larvae brains were dissected and stained for pH3 (also known as phospho-His3) (Fig. 5A-B′). Similar to controls, *Alk^Y1355S^* mutants exhibited four pH3-positive MB NBs, indicating that there are no detectable defects in NB quiescence. Later, during early pupal development, NBs shrink, exit the cell cycle and terminally differentiate (Homem et al., 2014), and in adult flies there is no detectable NB proliferation. To exclude the possibility that NBs continue to proliferate at later stages in *Alk^Y1355S^* mutants, we measured NB size in pre-pupae of control and *Alk^Y1355S^* in comparison to wL3 larvae. Similar to control, *Alk^Y1355S^* NBs undergo a progressive size reduction in pre-pupal stages indicating proper terminal differentiation (Fig. S8A,B). Finally, we did not detect excessive proliferation in adult brains of *Alk^Y1355S^* mutants using anti-pH3 staining (Fig. S8C). Taken together, no perturbations in NB quiescence were observed in *Alk^Y1355S^* mutants in first instar (L1), excluding this mechanism as the source of the observed hyperplasia. In addition, no change in proliferation dynamics could be detected at adult stages.

### *Alk* mutants display reduced levels of apoptosis

In models of childhood neuroblastoma, expression of the *MYCN* oncogene in the developing neural crest drives tumorigenesis. In a zebrafish neuroblastoma model, the additional transgenic expression of the *Alk^F1174L^* activated variant overcomes excessive apoptosis observed in MYCN-only tumors, decreasing cell death and promoting tumorigenesis (Zhu et al., 2012), suggesting an important role for Alk signaling in promotion of survival. Moreover, the zebrafish Alk family RTK Leukocyte tyrosine kinase (Ltk) that functions in neural crest-derived iridiophore development has been reported to promote survival when carrying a neuroblastoma-associated mutation (Fadeev et al., 2016). Further, in the fly visual system, Alk has been shown to be important for the survival of L3 neurons (Pecot et al., 2014), prompting us to address apoptosis in more detail. Indeed, Gene Ontology (GO) analysis of cluster 1 in our scRNA-seq dataset identified an enrichment of ‘cell-death-defective’ markers in postembryonic *Alk^Y1355S^* mutant brains (Fig. 5C). These included the Diap1 suppressor of apoptosis, which was also upregulated in bulk RNA-seq of *Alk^Y1355S^* brains (Fig. 5D,E). This prompted us to test whether decreased apoptosis in *Alk^Y1355S^* and *Alk^F1251L^* mutant brains may lead to the observed increased numbers of neurons. To investigate this, Cleaved caspase-1 (Dcp-1; also known as DCP1) antibody staining of *Alk^Y1355S^*, *Alk^F1251L^* and *Alk^ΔRA^* wL3 brains was performed, and Dcp-1-positive cells in the central brain area were counted. Significantly reduced numbers of Dcp-1-positive cells were observed in both *Alk^Y1355S^* and *Alk^F1251L^* mutants, although we did not observe increased apoptosis in *Alk^ΔRA^* mutant larval brains (Fig. 5F). The Dcp-1 analysis was complemented with two additional apoptosis assays – first, a terminal deoxynucleotidyl transferase dUTP nick end labeling (TUNEL) assay and, second, a PARP1 reporter – which both confirmed reduced levels of apoptosis in *Alk^Y1355S^* mutants (Fig. 5G,H). Finally, we analyzed apoptosis in the controlled setting of the Engrailed-positive anterior cluster (AC), posterior cluster (PC), medial cluster (MC)1 and MC2 hemi-lineages, in which immediate progeny of specific GMCs are removed via apoptosis (Kumar et al., 2009). In the MC2 lineage, all Engrailed-positive neurons are removed via apoptosis, while Engrailed-positive or -negative neurons in the MC1 lineage do not undergo programmed cell death (Kumar et al., 2009). In the AC and PC lineage, the Engrailed-negative GMC progeny are removed via apoptosis (Kumar et al., 2009). Analysis of the overall volume of the MC lineage via Engrailed staining, while simultaneously staining for Dcp-1, failed to identify any differences in MC lineage size in either *Alk^Y1355S^* or *Alk^ΔRA^* compared with control (Fig. 5I). However, analysis of Dcp-1-positive cells within and in proximity to the other hemi-lineages identified significantly more Dcp-1-positive cells around the AC lineage in *Alk^ΔRA^* compared to control and *Alk^Y1355S^.* This finding was supported by the presence of significantly fewer Dcp-1-positive neurons around the PC lineage in *Alk^Y1355S^* compared to control and *Alk^ΔRA^* (Fig. 5J). Taken together, these four independent assays support decreased levels of apoptosis in *Alk^Y1355S^* mutants as a contributory factor to the increased numbers of surviving neurons observed.

### Perturbed neuronal fate in the MB lineage in *Alk^Y1355S^* mutant brains

**Fig. 6.**
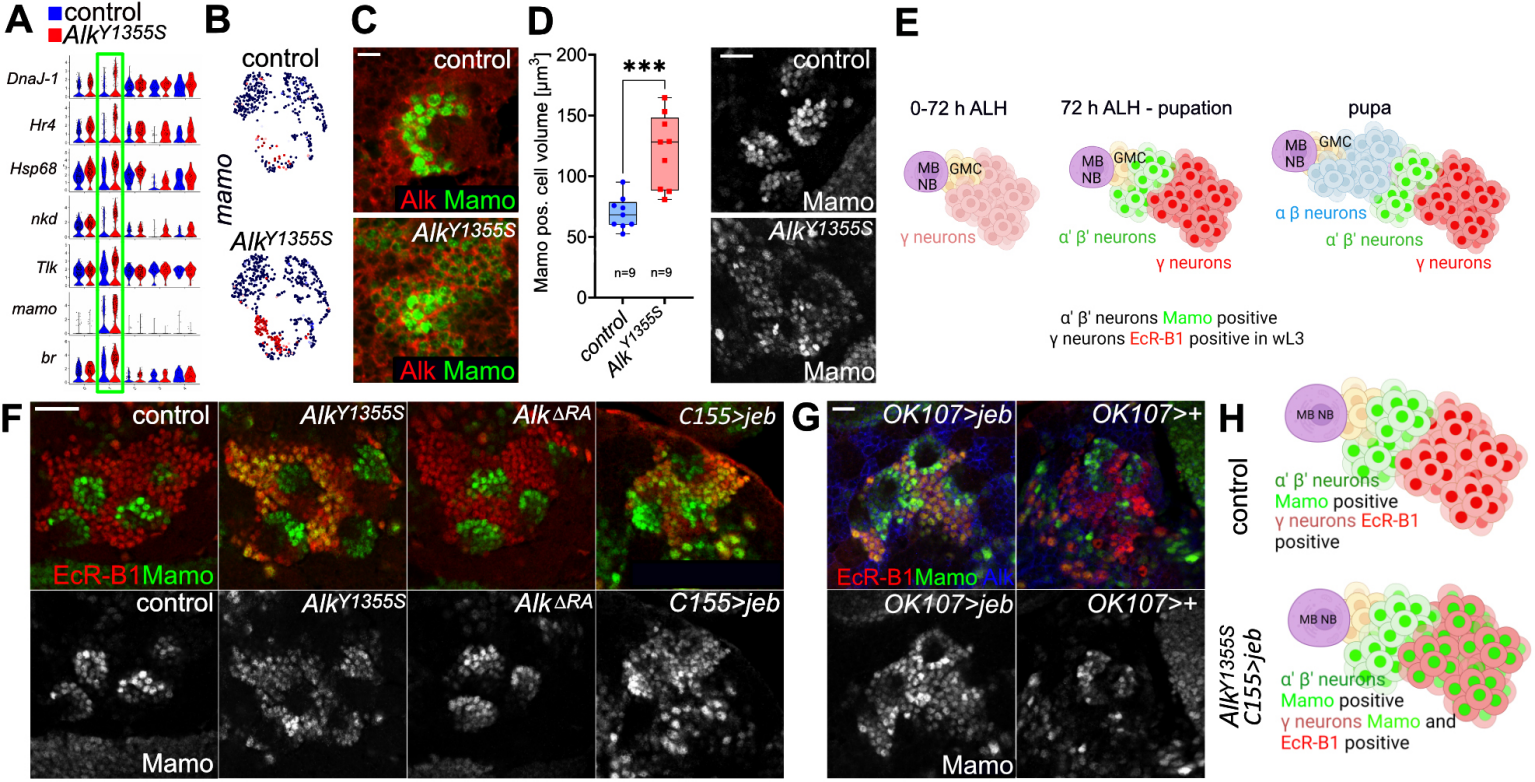
**Ectopic Mamo expression in the mushroom body (MB) γ-lineage in *Alk^Y1355S^* indicates perturbed α′β′- and γ-neuronal differentiation.** (A) Violin plots indicating mRNA expression of *mamo*, *br*, *Tlk*, *nkd*, *DnaJ-1*, *Hr4* and *Hsp68* in cluster 1 of the *Alk^Y1355S^* scRNA-seq dataset. (B) Feature plots projecting *mamo* mRNA expression in both wild-type and *Alk^Y1355S^* scRNA-seq datasets. (C) Mamo (green) and Alk (red) are co-expressed in neurons. Scale bar: 5 µm. *n*>10 larval brains. (D) Quantification of Mamo-positive cell area in homozygous *Alk^Y1355S^* compared to controls. In box plots, the box represents the 25-75th percentiles, and the line indicates the median; whiskers indicate minimum and maximum values. Representative examples are shown. Unpaired two-tailed Student's *t*-test; ***P<0.001. *n*=number of brain lobes analyzed. Scale bar: 20 µm. (E) Schematic showing the generation of the three different major MB lineages during development (adapted from Lee et al., 1999; Liu et al., 2019). Schematic created with biorender.com. This image is not published under the terms of the CC-BY license of this article. For permission to reuse, please see Lee et al. (1999). GMC, ganglion mother cell. (F) Mamo is detected in the MB γ-neuron lineage (EcR-B1 positive) in homozygous wL3 *Alk^Y1355S^* larvae, but not in controls or the homozygous *Alk^ΔRA^* mutant. Overexpression of Jeb ligand (*C155/+>jeb/+*) phenocopies the ectopic Mamo expression in the γ-lineage observed in *Alk^Y1355S^*. Scale bar: 20 µm. *n*>brain lobes analyzed per genotype in Fig. S9B. (G) MB-specific *OK107-Gal4* driver overexpressing Jeb (*OK107/+>jeb/+*) phenocopies the *Alk^Y1355S^* phenotype. Scale bar: 20 µm. *n*>brain lobes analyzed per genotype in Fig. S9B. (H) Schematic summarizing the ectopic Mamo expression observed in homozygous *Alk^Y1355S^* and *C155/+>jeb/+* compared to control MB lineages. Schematic created with biorender.com. scRNA-seq data from 45 dissected wL3 larval brains were used for A and B.

Interestingly, scRNA-seq analysis highlighted increased proportions of cells that exhibited high levels of expression of neuronal lineage markers such as *mamo*, *br*, *Tlk*, *nkd*, *DnaJ-1*, *Hr4* and *Hsp68* in cluster 1 of *Alk^Y1355S^* larval brains (Fig. 6A,B). To visualize differentially expressed markers, we analyzed the expression of the BTB/POZ-containing C2H2 zinc finger transcription factor Mamo via antibody staining in control and *Alk^Y1355S^*. Mamo has been reported to be critical for temporal specification of MB α′β′-neurons (Liu et al., 2019; Rossi and Desplan, 2020). Initial investigation confirmed that Mamo is expressed in Alk-positive larval neurons in the MB lineage (Fig. 6C). As our scRNA-seq datasets detected significantly more *mamo* expression in the *Alk^Y1355S^* mutant allele (Fig. 6A,B), we measured the overall volume of Mamo-positive cell clusters within the MB lineage, observing an increase in the total volume of Mamo-positive cells in *Alk^Y1355S^* (Fig. 6D) compared to the control, validating our scRNA-seq data.

To examine Mamo expression in *Alk^Y1355S^* larval brains further, we focused on the MB NB lineage in wL3. The MB lineage comprises three major cell types: early-born γ-neurons, middle-born α′β′-neurons and late-born αβ-neurons (Fig. 6E) (Lee et al., 1999). Mamo expression is restricted to α′β′-neurons, whereas EcR-B1 is exclusively expressed within γ-neurons in wL3 (Fig. 6E) (Lee et al., 2000; Liu et al., 2019; Rossi and Desplan, 2020). Remarkably, we observed ectopic expression of Mamo in EcR-B1-positive γ-neurons in *Alk^Y1355S^* mutant wL3 brains (Fig. 6F,H). To confirm that excessive Alk activity results in improper Mamo expression, we ectopically overexpressed Jeb with *C155-Gal4*, which also resulted in ectopic expression of Mamo in EcR-B1-positive MB γ-neurons (Fig. 6F,H). Analysis of Mamo in *Alk^ΔRA^* mutants showed that although increased Alk activation leads to Mamo expression, Alk is not critically required for normal Mamo expression in the α′- and β′-lineage, as Mamo expression in *Alk^ΔRA^* mutants MB α′β′-neurons is unchanged (Fig. 6F). These findings were confirmed by expression of Jeb using the MB-specific *OK107-Gal4* and *201γ-Gal4* drivers, which also led to Mamo expression in wL3 EcR-B1-positive MB γ-neurons (Fig. 6G; Fig. S9, quantification in Fig. S9B).

We next asked whether ectopic Mamo expression leads to a neuronal fate change in γ-neurons in *Alk^Y1355S^* larval brains that is maintained in adults, comparing axonal morphology and the MB cell bodies (MBCs) in wild-type and *Alk^Y1355S^* with antibodies to the Rho guanine exchange factor (RhoGEF) Trio. Trio is important for directional extension of neurites in the MB and is strongly expressed in the cytoplasm and plasma membrane of α′β′-cell bodies, whereas in γ-cell bodies expression is enriched at plasma membranes but largely absent from the cytoplasm (Awasaki et al., 2000). We also examined the Abrupt zinc finger BTB domain-containing transcription factor that functions in motoneuron guidance and connectivity (Hu et al., 1995).

**Fig. 7.**
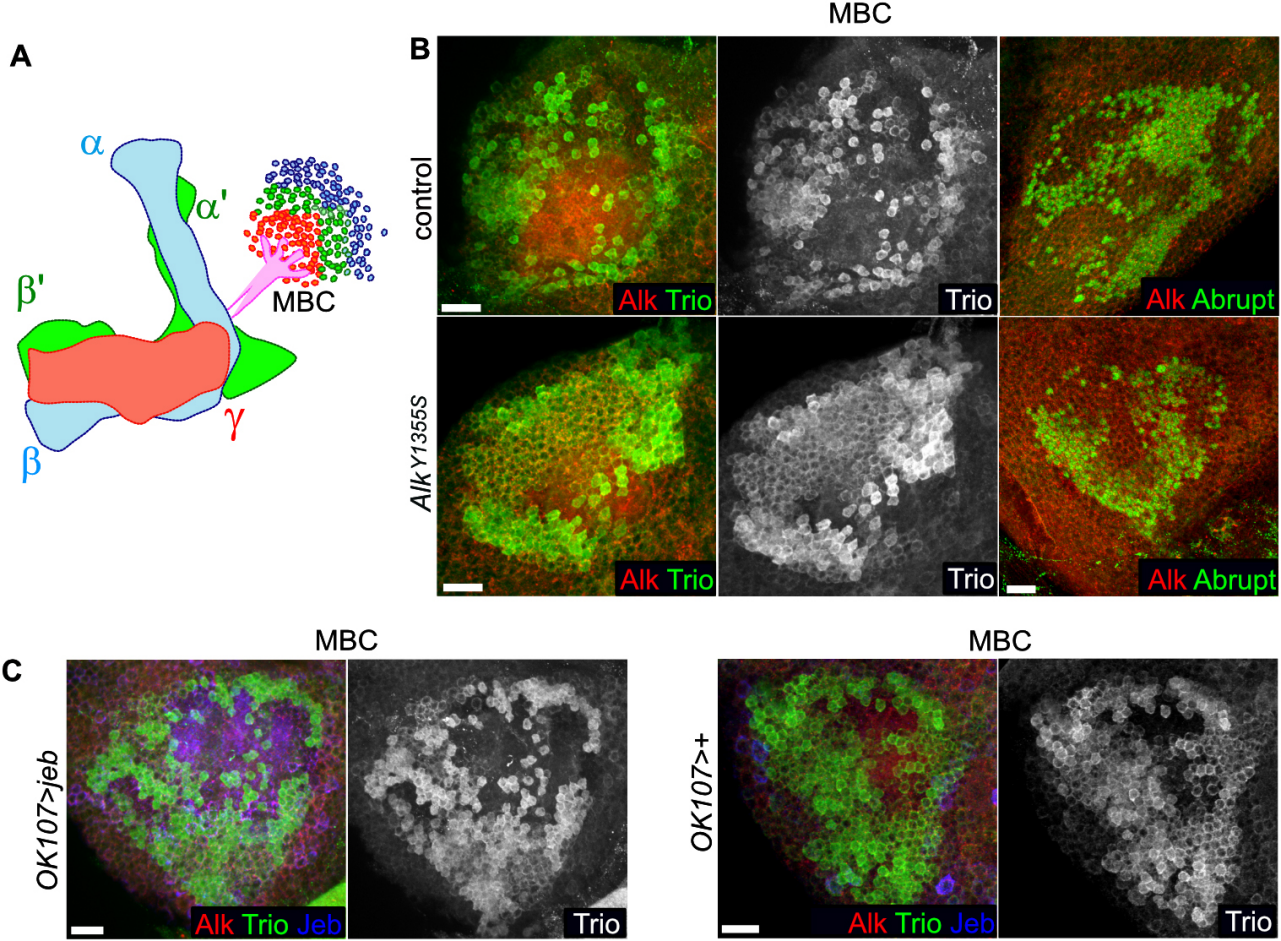
**Perturbed neuronal differentiation in *Alk^Y1355S^* is maintained to adulthood.** (A) Schematic displaying the three main MB neuronal types (αβ, α′β′ and γ) and the mushroom body cell bodies (MBC) in adults. (B) Analysis of Trio and Abrupt in the MBC of homozygous *Alk^Y1355S^* and controls. Scale bars: 20 µm. (C) Trio expression in MBC of *OK107/+>jeb/+* and *OK107/+* controls. Scale bars: 10 µm.

In adult flies, the γ-, α′β′- and αβ-MBCs branch anteriorly through the peduncle into three main lobes: the bifurcating α′β′- and αβ-lobes, and a single γ-lobe (Fig. 7A) (Crittenden et al., 1998). No gross defects in overall MB morphology were observed in *Alk^Y1355S^* adult brains (Fig. S10); however, examination of Trio and Abrupt expression in MBCs identified more cell bodies with enhanced and cytoplasmic Trio expression (Fig. 7B), whereas expression of Abrupt was decreased in *Alk^Y1355S^*. These results were strengthened by increased cytoplasmic Trio-positive cell bodies in *OK107>jeb* adults (Fig. 7C), further indicating that increased Alk signaling shifts γ identity to a more α′β′ neuronal identity in larval stages, which is maintained through metamorphosis into adulthood.

Taken together, our data show that neuroblastoma-associated Alk point mutations do not result in changes in NB number, or in gross increases in proliferation, but rather perturb neuronal fate specification, which leads to precocious Mamo expression in the γ-cell lineage of *Alk^Y1355S^* mutant wL3 brains that persists until adult stages.

## DISCUSSION

This work set out to address mechanisms by which Alk mutations affect neural development. This is particularly relevant in pediatric neuroblastoma, where patients with *ALK* mutations exhibit poor prognosis, and tumors are considered to arise due to defective differentiation during development of the nervous system. For this reason, we generated and characterized two independent *Alk* mutations (*Alk^Y1355S^* and *Alk^F1251L^*) in *Drosophila*, for which the human orthologs (*ALK^Y1278S^* and *ALK^F1174L^*) have previously been characterized as ‘gain-of-function’ mutations in neuroblastoma patients (Chand et al., 2013; Guan et al., 2017).

Flies harboring the *in locus* modifications *Alk^Y1355S^* or *Alk^F1251L^* are homozygous viable, which is in line with observations in vertebrate model systems in which Alk gain-of-function mutations have been investigated (Borenäs et al., 2021; Cazes et al., 2014; Fadeev et al., 2016). Employing *Drosophila* allowed us to investigate neurogenesis in a highly controlled environment, revealing, first, that *Alk^Y1355S^* and *Alk^F1251L^* mutant brains exhibit mild neuronal hyperplasia and, second, no increase in NB numbers. This is in keeping with reports of hyperplasia in peripheral nervous system ganglia of *Alk* ‘gain-of-function’ mice (Borenäs et al., 2021; Cazes et al., 2014; Ono et al., 2019). Interestingly, although the mechanisms underlying this neuronal hyperplasia are not well understood, it is clear that mutations in the endogenous *Alk* locus collaborate with the *MYCN* oncogene to drive highly penetrant and aggressive neuroblastoma in mice (Borenäs et al., 2021; Cazes et al., 2014; Ono et al., 2019).

Interestingly, reduced apoptosis leads to hyperplasia in the nervous system of transgenic zebrafish expressing Alk gain-of-function variants (Zhu et al., 2012), and regulation of apoptosis during development is important to establish final neuron numbers in the CNS in *Drosophila* (Pinto-Teixeira et al., 2016). Indeed, our results reveal decreased apoptosis as a contributing mechanism underlying the hyperplastic phenotype observed in *Alk^Y1355S^* and *Alk^F1251L^* brains. Additional reports suggest that Alk signaling leads to advantages in growth and cell competition (Wolfstetter et al., 2020), and Alk has been shown to promote L3 neuronal survival in the *Drosophila* optic lobe (Pecot et al., 2014). Further, knockdown or inhibition of Alk in zebrafish induces apoptosis in the fish hindbrain (Yao et al., 2013).

One important aspect is the expression of *Alk* itself. Our scRNA-seq datasets identified strong expression of *Alk* mRNA in neuronal lineages particularly in mature neurons, but not in NBs, and this was confirmed at the protein level by immunostaining. This Alk expression profile is in line with an anti-apoptotic effect in neurons later during the differentiation process. Whether *Alk^Y1355S^* mutants are protected from apoptosis throughout development and in the adult brain is not known. However, in zebrafish, aberrant signaling by the Alk family RTK, Ltk, which carries a neuroblastoma-related mutation (*Ltk^moonstone^*), promotes survival of neural crest-derived iridophores and increases their final number (Fadeev et al., 2016). In addition, our findings that *Alk^Y1355S^* and *Alk^F1251L^* do not drive uncontrolled proliferation in the *Drosophila* brain support earlier reports in mouse and zebrafish models (Borenäs et al., 2021; Cazes et al., 2014; Fadeev et al., 2016; Ono et al., 2019), where mutation of *Alk* alone is insufficient to drive spontaneous tumor development.

An important tool generated in this study is the *Alk^ΔRA^* mutant in which *Alk* mRNA and protein expression in the larval CNS is strongly reduced. Analysis of *Alk^ΔRA^* suggests that Alk activity is not critical for neuronal differentiation in the MBs. Our observation of increased apoptosis in this *Alk^ΔRA^* mutant, although slight, is in keeping with the finding of decreased apoptosis in *Alk^Y1355S^* and *Alk^F1251L^* brains. Whether higher levels of apoptosis might be observed in conditions of stress or injury in the *Alk^ΔRA^* mutant will be interesting to study in the future.

Our results also produced several surprising findings. One of these was that neither *Alk^Y1355S^* nor *Alk^F1251L^* was able to rescue founder cell specification in the developing VM in the absence of Jeb, suggesting that these activating *Alk* alleles are still ligand dependent. The issue of ligand dependency has not been tested in either mouse, zebrafish or human systems, in which ALK is activated by the ALKAL ligands (Fadeev et al., 2018; Guan et al., 2015; Mo et al., 2017; Reshetnyak et al., 2015). Although it is clear that both *Alk^Y1355S^* and *Alk^F1251L^* are ligand dependent in the developing fly VM, we do not have the tools to test this in the *Drosophila* brain and therefore cannot be sure if the observed ligand dependence is tissue specific. Nonetheless, this finding has interesting and important implications for neuroblastoma. Although human *ALK-F1174L* and *ALK-Y1278S* (orthologous to *Drosophila Alk^F1251L^* and *Alk^Y1355S^*) are classified as ligand-independent mutations (Chand et al., 2013), we cannot exclude that these human ALK mutations still require ligands to initiate activation at endogenous levels of expression. Thus, ALK ligands might still be important for tumor formation and progression, even in the case of activating ALK mutations.

The use of scRNA-seq analysis, in addition to bulk RNA-seq, was critical to identify apoptotic and neuronal differentiation defects during development in *Alk^Y1355S^* mutants, revealing potential Alk signaling targets including *mamo*, a BTB transcription factor that defines terminal identity in certain MB neurons. In *Drosophila*, the MB is composed of ∼2000 neurons of the three main neuronal subtypes: γ, α′β′ and αβ (Cognigni et al., 2018; Ito et al., 1997; Lee et al., 1999). γ-, α′β′- and αβ-neurons are produced sequentially during defined developmental periods in response to Imp and Syp gradients (Liu et al., 2015; Ren et al., 2017; Syed et al., 2017a; Yang et al., 2017). Elegant work has shown that terminal identity and maintenance of MB α′β′-neurons is established by Mamo expression (Liu et al., 2019), which depends on extrinsic receptor signaling via the activin receptor Babo (Rossi and Desplan, 2020). Although we did not identify neuronal differentiation defects in *Alk^ΔRA^* MBs, the γ-neuron lineage was Mamo positive upon pan-neuronal expression of Jeb and in *Alk^Y1355S^* wL3 brains. Thus, aberrant Alk activity leads to inappropriate Mamo expression in the γ-lineage at this stage, and perturbation of MB neuronal fate that persists to adult stages, where MB neuron cell bodies exhibit expanded Trio expression. Interestingly, Alk signaling has previously been linked to learning and memory (Gouzi et al., 2011; Weiss et al., 2017, 2012), which could be caused by fate perturbations in MB neuron lineages.

Together, the results presented here identify neuronal hyperplasia caused by decreased apoptosis in *Alk^Y1355S^* and *Alk^F1251L^* mutant brains. scRNA-seq identified molecular neuronal differentiation defects that were validated in the well-characterized process of MB neuron development. To our knowledge, this is the first time that molecular defects in neuronal differentiation have been identified in response to *Alk-*activating mutations. These findings have potentially important implications for pediatric neuroblastoma, where mutation of human ALK at orthologous sites (*ALK-Y1278S* and *ALK-F1174L*) is associated with tumor progression in the developing peripheral nervous system.

## MATERIALS AND METHODS

### *Drosophila* husbandry

Standard *Drosophila* husbandry procedures were followed (Ashburner, 1989). *Drosophila* stocks were reared on standard diet at room temperature. Crosses were performed at 25°C, 60% humidity and 12 h/12 h day–night cycle.

### Fly stocks

Fly stocks used were obtained from the Bloomington *Drosophila* Stock Center (NIH P40OD018537): *P{GawB}elav^C155^ (C155-Gal4)* (BL-458), *y^1^, {Mvas-Cas9}ZH-2A, w^1118^* (BL-51323; used as wild-type control), *w^1118^; P{70FLP}10* (BL-6938), *w^1118^; P{GAL4-Act5C(FRT.CD2).P}S* (BL-4780), *P{UAS-RFP.W}3/TM3, Sb^1^* (Bl-30558), w*; P{Ubi-p63E(FRT.STOP)Stinger}15F2 (BL-32251), y^1 *^; P{UAS-CD8::PARP1-Venus}3 (BL-65609), w*; P{GawB}OK107 ey^OK107^/In(4)ci^D^, ci^D^ pan^ciD^ sv^spa-pol^ (BL-854*)*, *y^1^ w^67c23^; P{UAS-mCD8::GFP.L}LL5 P{GawB}Tab2^201Y^* (BL-64296). Other lines used were as follows: *P{UAS-Alk.EC.MYC}* (Bazigou et al., 2007), *P{UAS-jeb}* (Varshney and Palmer, 2006), *jeb^weli^* (Stute et al., 2004), *UAS-hALK.F1174L* and *UAS-hALK* (Martinsson et al., 2011), *UAS-hALK.Y1278S* (Guan et al., 2017), *UAS-GFP.caax* (Finley et al., 1998), *TI{TI}Alk^KO^* (Wolfstetter et al., 2017).

### Generation of *Alk* mutations employing CRISPR/Cas9-mediated genome editing

The *Alk^F1251L^* and *Alk^Y1355S^* alleles were generated using CRISPR/Cas9-induced HDR (Gratz et al., 2013). sgRNA target sites were identified using the flyCRISPR target finder (https://flycrispr.org/) (Gratz et al., 2014). Two sgRNAs for each approach (sgRNA1 Y1355S: TCGCCGATTTTGGCATGTCCCGG; sgRNA2 Y1355S: TCGGACTACTATCGCAAGGGAGG; sgRNA1 F1251L: CCTGAAGGAGGCGGCCATAATGG; sgRNA2 F1251L: CAAGTTCAATCACCCGAATATGG) were cloned into *pBFv-U6.2* expression vector (Genome Engineering Production Group at Harvard Medical School). HDR donor vectors were generated by Genescript. Design of the *Alk^Y1355S^* and *Alk^F1251L^* donor constructs was as follows: *Y1355S*: containing silent mutations to prevent annealing and destruction of the donor by Cas9: TtGCtGAcTTcGGtATGTCaCG and TCaGAtTAtTAcCGtAAaGGtGG; homology arms: 507 bp upstream, 506 bp downstream of the desired mutation site (Fig. S11). *F1251L*: silent mutations plus integrated nucleotide exchange to create the desired mutation: CTaAAaGAaGCaGCaATcATGGCaAAGcTtAAcCAtCCaAAcATG; homology arms: 1052 bp upstream, 902 bp downstream (Fig. S12). sgRNAs and donor constructs were injected into *y^1^, {Mvas-Cas9}ZH-2A, w^1118^* embryos by BestGene Inc. Positive CRISPR/Cas9 mutants were identified by PCR screening of single males using primers directed against the region of HDR-integrated silent mutations (forward primer, 5′-ATCCTAATGATCTCGCTTGCCGTG-3′; Y1355S reverse primer, 5′-CTcCCGATCaGAtTAtTAcCGtAAaG-3′; F1251L reverse primer, 5′-TTtGGaTGgTTaAgcTTtGCCATgAT-3′) and subsequent sequencing (Eurofins Genomics).

To generate the kinase-dead *Alk^D1347A^* mutant allele and the *Alk^D1347A, Y1355S^* double mutant, the *Alk^Y1355S^* HDR donor construct was modified accordingly using a Q5^®^ site-directed mutagenesis kit (NEB, E0554S). Owing to the proximity of both codons within the sequence, the guide RNA target sequences (sgRNA Y1355S nr.1 and nr.2, cloned into *pU6-BbsI-chiRNA*) (Gratz et al., 2013) were used for injection into *y^1^, {Mvas-Cas9}ZH-2A, w^1118^* embryos. Positive candidates were identified by single-fly PCR screening with Y1355S forward and reverse primers (*Alk^D1347A, Y1355S^*) or lethality screening (*Alk^D1347A^*) and further sequenced (Eurofins Genomics) (Fig. S13). All positive candidates obtained failed to complement the *Alk^KO^* kinase domain deletion mutation.

The *Alk^ΔRA^* mutant allele was created by CRISPR/Cas9-induced non-homologous end joining in a dual-guide approach (sgRNA10_RA, 5′-CCGTCTATCCGCGATTCTGAGGG-3′; sgRNA20_RA, 5′-GCGACAGTGGCGCACTCTGGCGG-3′) targeting the 5′UTR of the *Alk-RA* isoform. sgRNA sequences were cloned into the *pBFv-U6.2* expression vector (Genome Engineering Production Group at Harvard Medical School) and the subsequent constructs injected into *y^1^, {Mvas-Cas9}ZH-2A, w^1118^* embryos by BestGene Inc. Screening of deletion events was performed on F2 single males by PCR using primers flanking the intended deletion (forward primer, *Alk-RA* Fw1, 5′-cgaaatttttcctgcagctc-3′; reverse primer, *Alk-RA* Rv1, 5′-atggggtccttaatgcactg-3′), and deletions were further characterized by sequencing (Eurofins Genomics) (Fig. S14).

### Pupal size measurement

Six virgin females and four males of each genotype were reared for 2 days on a standard diet at 25°C. The flies were removed, and the vials were kept at 25°C. Shortly before hatching, female pupae were scored and collected on double-sided sticky tape on a glass slide, briefly frozen at −20°C and analyzed with a ZEISS AxioZoom V16 stereo microscope.

### Fixation and immunohistochemistry staining of embryos

Embryo staining was carried out as described previously (Muller, 2008). For pERK staining, the protocol from Gabay et al. (1997) was followed.

### Fixation and immunohistochemistry staining of larval and adult brains

Brains of all stages were dissected in PBS, collected on ice in PBS containing 3.7% formaldehyde, and subsequently fixed in the same solution for 30 min at room temperature (RT), followed by permeabilization in PBS containing 1% Triton X-100 for 10 min and three washes in PBS containing 0.5% Triton X-100 (PBT). Samples were blocked in 5% goat serum (Jackson ImmunoResearch) in PBT, then primary antibody was added overnight at 4°C. Samples were washed four times in PBT and incubated in PBT containing the secondary antibody and DAPI for 2 h at RT followed by four washes in PBT. Samples were mounted in Fluoromount G and analyzed with a ZEISS Axio Imager.72 microscope. A ZEISS LSM800 confocal microscope and ZEN 3.5 Blue edition software were used to acquire images. Acquired images were analyzed with ImageJ version 1.53c and assembled using Affinity Photo and Designer version 1.10.0.1127.

### Antibodies

Primary antibodies used were as follows: guinea pig (gp)-anti-Alk (1:1000) and rabbit (rb)-anti-Alk (1:1000) (Lorén et al., 2001), gp-anti-Jeb (1:1500) (Englund et al., 2003), mouse (m)-anti-activated MAPK/dephosphorylated ERK1/2 (1:200, Sigma-Aldrich, M8159), rb-anti-pH3 (1:500, Millipore, 0657C), rb-anti-Miranda (1:1000, Abcam, ab197788), chicken (ch)-anti-GFP (1:500, Abcam, ab13970), rb-anti-RFP (1:1000, Abcam, ab62341), rb-anti-cleaved PARP1 (1:50, Abcam, ab2317, anti-β-Galactosidase (1:150, Abcam, ab616), rb-anti-Tubulin (1:5000; Cell Signaling Technology, 11H10) and rb-anti-Dcp-1 (1:50; Cell Signaling Technology, 9578), as well as m-anti-Dlg (1:500; 4F3), rat-anti-Elav (1:100; 9F8A9), m-anti-EcR-B1 (1:100; ADA4.4), m-anti-Prospero (1:100; MR1A), m-anti-Trio (1:100; 9.4A), m-anti-Abrupt (1:50) and m-anti-Engrailed (1:50; 4D9) from Developmental Studies Hybridoma Bank. Guinea pig-anti-Mamo (1:100) was a gift from Claude Desplan’s laboratory (Rossi and Desplan, 2020); rb-anti-Ase (1:400) was a gift from Cheng-Yu Lee’s laboratory (Weng et al., 2010). Secondary antibody goat anti-rb HRP (Invitrogen, 11859140) was used for immunoblotting; other secondary antibodies were purchased from Jackson ImmunoResearch. DAPI (1 mg/ml) (Sigma-Aldrich, D9564-10MG) was used at 1:1000.

### *In situ* hybridization

Whole-mount *in situ* hybridization was carried out as described previously (Lécuyer et al., 2008) with adaptations according to Pfeifer et al. (2014).

### HCR v.3

The HCR probe sets were generated by Molecular Instruments (www.molecularinstruments.com) against full-length *Alk* and *jeb* mRNA sequences from the NCBI database (accession numbers NM_001274098.1 and NM_136882, respectively). HCR amplifiers were as follows: *jeb*, B3 with the A488 amplifier fluorophore; *Alk*, B2 with the 546 amplifier fluorophore. Fluorescent *in situ* hybridization was carried out according to the protocol for *Drosophila* embryos for HCR v.3 from Molecular Instruments with the following modifications: Proteinase K digestion was carried out with 0.01 mg/ml proteinase K in PBT for 10 min.

### Immunoblotting

Brains from 30 wL3 larvae were dissected, collected on ice and subsequently lysed in RIPA buffer. Protein concentration was measured using a BCA Protein Assay Kit (Thermo Fisher Scientific). Laemmli buffer (final concentration 1×) was added prior to loading on a 7.5% SDS-PAGE gel. After transfer to PVDF (Millipore, IPVH00010), membranes were immunoblotted with primary antibodies overnight at 4°C. Primary antibodies used were gp-anti-Alk (1:3000) (Lorén et al., 2001) and rb-anti-tubulin (1:5000; Cell Signaling Technology, 11H10). After washing, membranes were incubated with rb-anti-HRP (Invitrogen, 11859140) secondary antibodies at RT for 1 h. ECL prime (Thermo Fisher Scientific, GERPN2236) was used for detection.

### Clonal analysis

To generate RFP or GFP clones, *hs-flp* females were crossed to males carrying *Act5c-Gal4>FRT.CD2>UAS-RFP* or *Ubi-p68E>FRT.stop>stinger* in either control or *Alk^Y1355S^* backgrounds. L1 larvae were collected from 2 h collection plates after hatching and grown at 25°C for 72 h. Larvae were subsequently heat shocked at 37°C for 10 min and reared at 25°C for a further 24 h.

### EdU pulse and chase experiments

The Click-iT™ EdU Alexa Fluor™ 488 Imaging Kit from Thermo Fisher Scientific (C10329) was employed according to the manufacturer's instructions. L3 larvae were reared on 0.2 M EdU in potato-based fly food for 3 h (3 h pulse) for analysis of NB I, 1.5 h (1.5 h pulse) for NB II and dissected immediately afterwards, or reared for 24 h at 25°C (24 h chase, for NB I) and then dissected and fixed. Staining was carried out according to the manufacturer's instructions.

### TUNEL assay

ApopTag^®^ Fluorescein In Situ Apoptosis Detection Kit (Sigma-Aldrich, S7110) was employed according to the manufacturer's instructions.

### *In vitro* brain culture

wL3 larvae were washed twice in PBS and once in 70% ethanol before collection and dissection in Schneider's medium. Dissected brains were cultured in Schneider's medium containing 10% heat-activated fetal bovine serum (FBS; Sigma-Aldrich, F7524), 0.01% insulin solution (Sigma-Aldrich, I0516-5ML), 1% penicillin/streptavidin (HyClone, SV30010), 1 µg/ml 20-hydroxyecdysone (Sigma-Aldrich, H5142-5MG) for 18 h, 36 h or 120 h. Prior to fixation, brains were washed three times in PBS and then fixed in 4% formaldehyde in PBS for 20 min at RT. Staining was carried out as described above in the ‘Fixation and immunohistochemistry staining of larval and adult brains’ staining section.

### CNS dissociation for cell sorting and scRNA-seq analysis

Forty-five wL3 larvae per genotype were collected and washed in PBS. CNSs were dissected and collected in ice-cold PBS for a maximum of 1 h. After rinsing twice in ice-cold PBS, CNSs were incubated in 200 µl dissociation solution (1 mg/ml collagenase I, Merck, C9891) for 1 h with continuous agitation, after which the reaction was stopped by addition of 10 ml PBS [0.04% bovine serum albumin (BSA)] and left for 10 min to precipitate larger debris. The resulting dissociated cell solution was sieved through 70 µm and 40 µm cell strainers to remove cell clumps. The filtered suspension was centrifuged at 860 ***g*** for 5 min at 4°C. Supernatant was discarded, and cells were resuspended at 10^8^ cells/ml (or at least in 100 μl) PBS, 2% FBS and 1 mM CaCl_2_. Dead cells or debris from the dissociated samples were removed using an EasySep Dead Cell Removal (Annexin V) Kit (STEMCELL, 17899) according to the manufacturer's guidelines. The remaining cells were respectively labeled with aqua-fluorescent reactive dye (dying cells) and Calcein Violet AM (living cells) using a LIVE/DEAD Violet Viability/Vitality Kit (Molecular Probes, L34958) according to the manufacturer's guidelines. Finally, each sample was washed twice in PBS, 2% FBS and resuspended in 500 µl PBS, 2% FBS. Living cells were enriched using a FACSAria III cell sorter (BD Biosciences) based on LIVE/DEAD staining. Cells were sorted using an 85 µm nozzle into Eppendorf tubes that had been pre-coated with PBS containing 2% BSA.

### Single-cell library preparation, sequencing and scRNA-seq analysis

Approximately 7000 sorted cells were directly loaded in sheath fluid onto one lane of a Chromium 10x chip (10x Genomics), and libraries were prepared using the normal workflow for Single Cell 3´ v3 libraries (10x Genomics) and sequenced on the NextSeq 500 platform (Illumina). Initial preprocessing of scRNA-seq datasets began with demultiplexing, read QC, mapping (STAR aligner with the *Drosophila melanogaster* genome), and quantification of barcodes and unique molecular identifiers using the Cell Ranger (5.0) software. The wild-type dataset resulted in a total of 4081 cells, and the *Alk^Y1355S^* dataset resulted in a total of 4222 cells. Downstream analysis including cell preprocessing, count normalization, feature selection, integration, clustering, dimensionality reduction/projection, trajectory inference and differential expression testing was performed using the R-based pipeline Seurat (4.0.3) (Stuart et al., 2019) and the Python-based pipeline Scanpy (Wolf et al., 2018). Cell quality was assessed by the proportion of cells with unique feature counts between 200 and 5000 (removing poor-quality cells), and transcripts between 500 and 50,000 (removing low-quality transcripts) and with less than 20% mitochondrial genes. After preprocessing, 3967 and 4099 cells remained, with a total of 10,411 and 10,316 RNA features for the control and *Alk^Y1355S^* datasets, respectively. Normalization of scRNA-seq datasets and integration analysis were performed with the SCTransform approach (Hafemeister and Satija, 2019). The top 3000 highly variable genes were selected to determine the true dimensionality of the dataset. Principal component analysis was used for clustering, and, based on elbow plots, the numbers of principal components were determined. Non-linear dimensional reduction using UMAP, and neighborhood identification with k=30 was used. Clusters were identified using the Louvain algorithm (Blondel et al., 2008), with 0.56 resolution. To determine clusters, marker genes of each cluster were identified by FindAllMarkers function (Seurat) and logistic regression analysis (Scanpy). Based on canonical markers and gene expression profiles, genes in similar clusters were merged and annotated based on existing knowledge and literature (Ariss et al., 2020; Brunet Avalos et al., 2019; Cattenoz et al., 2016; Estacio-Gómez et al., 2020; Michki et al., 2021).

To identify differentially expressed genes, expression profiles of each cluster across *Alk^Y1355S^* and wild-type conditions were compared by the MAST approach (Finak et al., 2015), with a threshold of log fold change of average expression between the two groups ≥0.2 and *P*-value≤0.05. Cluster correlation was performed using Pearson correlation algorithms (Scanpy), and relationship between clusters was determined by unrooted-phylogenetic tree (Seurat). GO analysis was performed using the R package EnrichR (employing FlyEnrichr as reference) (Kuleshov et al., 2016) on cluster 1 marker genes identified from NB-enriched cells (*Alk^Y1355S^*).

### RNA-seq and analysis

L3 larvae (*n*=45) were collected and quickly washed in water to remove food and yeast. Larvae were placed in ice-cold PBS in a depression well. Larval CNSs were fine dissected and transferred into microcentrifuge tubes containing 250 μl of an ice-cold 1:1 solution of RNAse-free PBS and RNALater (Invitrogen, AM7020). CNS samples were centrifuged at 382 ***g*** for 5 min at 4°C, and the supernatant was removed. Then, 500 μl fresh RNALater solution was added, and samples were stored at −80°C. Once CNS collection for three biological replicates per genotype had been performed, RNA extraction was carried out according to the manufacturer's protocol (Promega ReliaPrepTM RNA Tissue Miniprep System, Z6111). RNA concentration was measured using NanoDrop OneC (Thermo Fisher Scientific), and RNA integrity was checked by agarose gel electrophoresis. Four to six micrograms of total RNA per biological replicate were sequenced (Novogene Co. Ltd). Samples were assessed for quality with an Agilent 2100 Bioanalyzer system, and paired-end sequencing was performed on an Illumina platform. Over 40 million reads/genotype were generated and mapped to the genome at a rate of 94-96%. *Drosophila melanogaster* (ensemble bdgp6_gca_000001215_4 genome assembly) was used. HISAT2 algorithm for alignment and DESeq2 R package (Anders and Huber, 2010) for differential gene expression were used.

### Alk domain sequence and structure analysis

Pairwise alignment of *Drosophila melanogaster* Alk and human ALK was performed using the EMBOSS Needleman-Wunsch algorithm (Madeira et al., 2019). The three-dimensional structure of the *Drosophila* Alk kinase domain was modeled based on the human ALK crystal structure dataset template (NCBI Protein Domain and Macromolecular Structures database, PDB ID 4TT7) and the annotated *Drosophila* Alk-PA sequence (NP_001261027.1) using PyMol 1.8.6.0 software.

### Statistical analysis

Data acquirements and volume calculation was carried out with Microsoft Office Excel software. Statistical analysis was performed with GraphPad Prism version 9.3.1 software.

## Supplementary Material

Supplementary information
