# Supplementary material for: Patient-associated mutations in Drosophila Alk perturb neuronal differentiation and promote survival
Source: Dis Model Mech. 2022 Aug 16;15(8):dmm049591. doi: 10.1242/dmm.049591 (PMC9403751; doi:10.1242/dmm.049591)
Supplement: Supplementary information [file dmm-15-049591-s1.pdf]

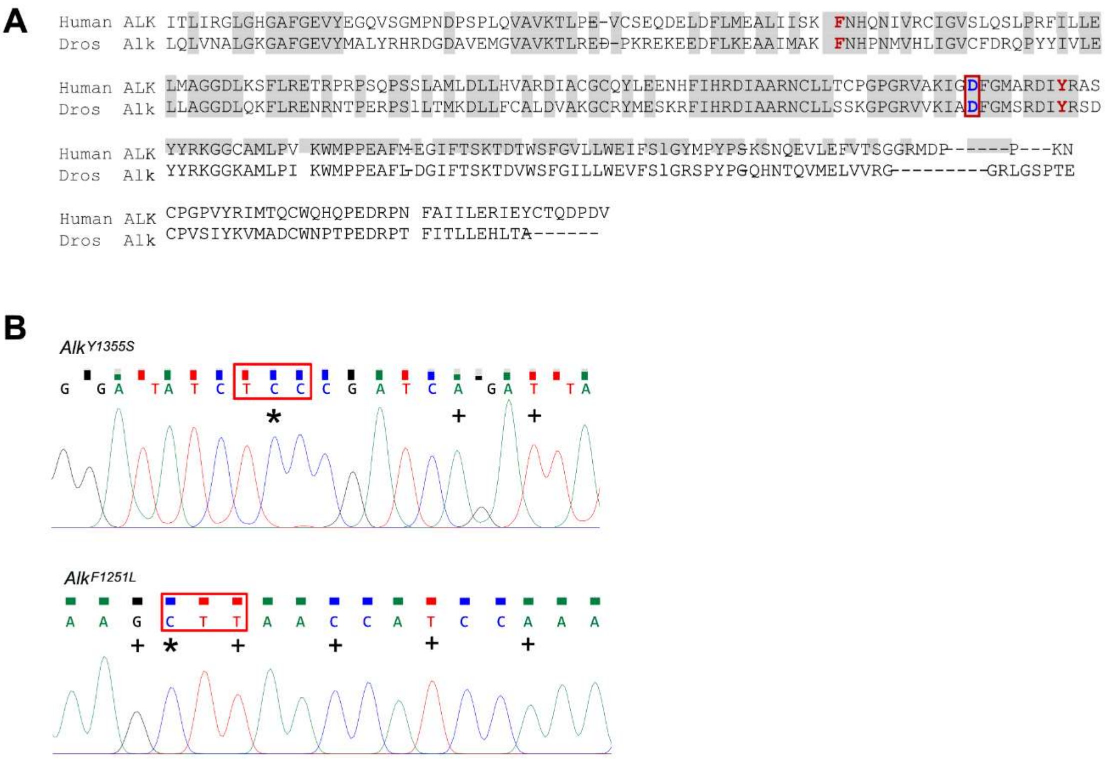

Fig. S1. Alignment of human and *Drosophila* Alk kinase domain and CRISPR/Cas9 mutant sequence

A Protein sequence alignment of human and *Drosophila* Alk in the kinase domain, with conserved regions highlighted in grey. Two mutated residues found in human neuroblastoma, F1174 and Y1278 (orthologous to *Drosophila* F1251 and Y1355, respectively), are indicated in red. D1268 in human ALK (orthologous to *Drosophila* D1345) residue predicted to be essential for kinase activity is indicated in blue with a red box.

B Chromatogram confirming CRISPR/Cas9-mediated nucleotide exchange (asterisks) in the homozygous *Alk*^{F1251L} and *Alk*^{Y1355S} mutant alleles, + indicates silent mutations.

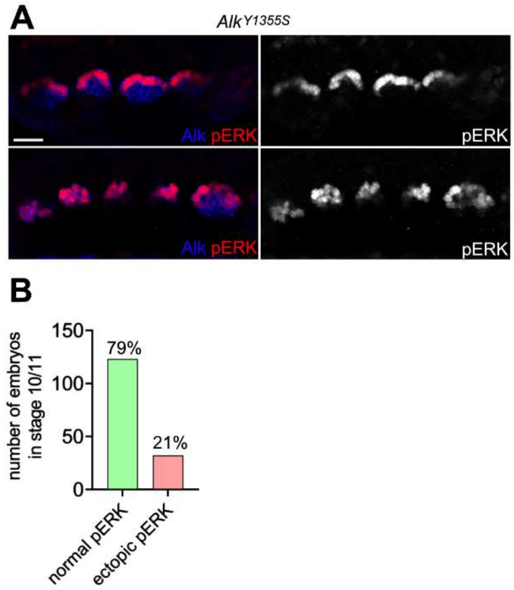

Fig. S2. Quantification of ectopic phospho-ERK in the visceral mesoderm in homozygous *Alk^{Y1355S}*.

A Analysis of phospho-ERK positive VM cells reveals variation of ectopic phospho-ERK in homozygous *Alk^{Y1355S}* ranging from a wildtype phospho-ERK pattern (shown in upper panels) to ectopic phospho-ERK in all VM cells (shown in lower panels) similar to Jeb ligand overexpression. Ectopic phospho-ERK pattern was also variable within VM clusters in the same animal.

B A total of 155 stage 10/11 embryos were analysed. Ectopic phospho-ERK was observed in 32 embryos (21%) examined.

Disease Models & Mechanisms • Supplementary information

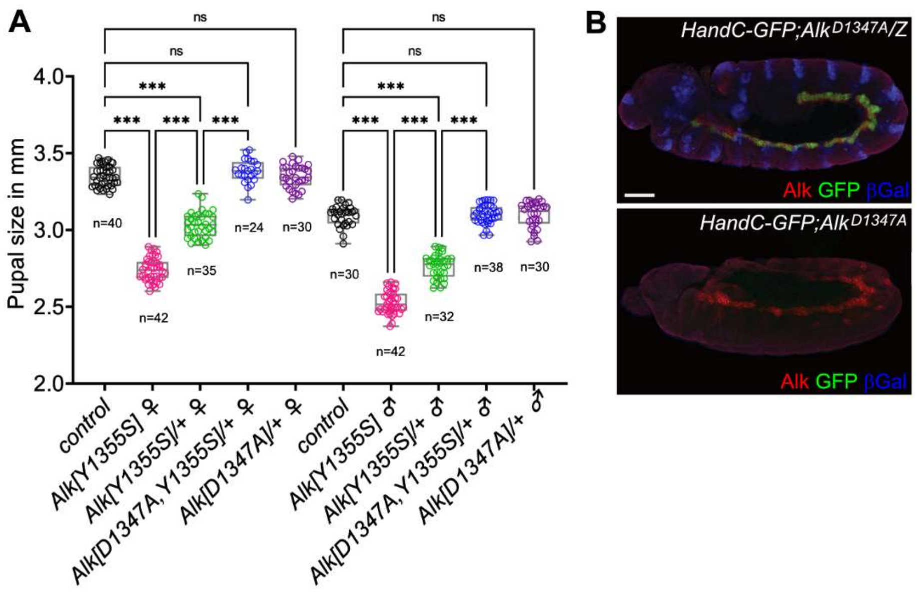

Fig. S3. Analysis of *Alk*^{D1347A} and *Alk*$^{D1347A,\ Y1355S}$.

A Pupal size analysis shows that the small pupal size phenotype of *Alk*^{Y1355S}/+ is fully rescued in *Alk*$^{D1347A,\ Y1355S}$/+. Ordinary one-way ANOVA, and Unpaired t test. $p<0.001$. n = animals analyzed.

B Homozygous *Alk*^{D1347A} mutants display an *Alk* loss of function phenotype in the VM as visualized by lack of phospho-ERK staining in FCs at stage 10/11. Scale bar = 50 µm. n > 150.

Disease Models & Mechanisms • Supplementary information

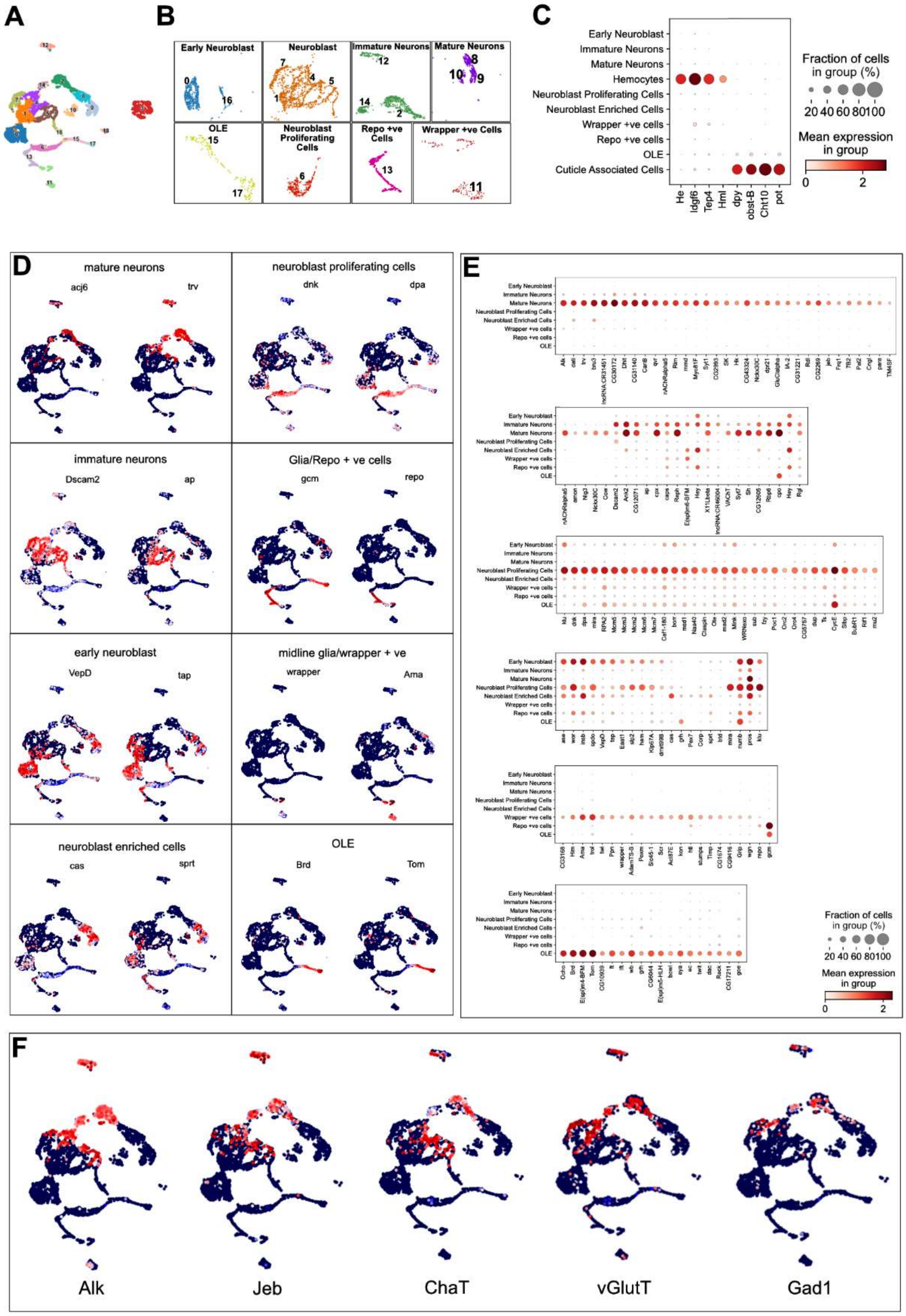

Disease Models & Mechanisms • Supplementary information

Fig. S4. Detailed analysis of scRNA-seq dataset annotation.

scRNA-seq data from 45 dissected and pooled wandering third instar (wL3) larval brains was used for A, B, C, D, E and F.

A UMAP displaying the initially defined 19 clusters from the wL3 scRNA-seq dataset.

B Similar clusters were merged to define the cellular heterogeneity, resulting in eight cell clusters (Early Neuroblast, Neuroblast Enriched Cells, Immature Neurons, Mature Neurons, Neuroblast Proliferating Cells, OLE, Repo +ve cells and Wrapper +ve cells) shown by UMAP.

C Dotplot showing the expression of canonical markers for the Hemocyte and Cuticle Associated Cell clusters.

D Feature plots visualizing a pair of canonical markers in the eight clusters.

E Dotplots indicating unique markers for the eight defined clusters. Color scale indicates mean expression (red gradient) and percentage of cells distributed (dot size).

F Feature plots showing the expression of *Alk, Jeb*, cholinergic (*ChAT*), glutamatergic (*VGlut*) and GABAergic neurons (*Gad1*) neurons in the wL3 scRNA-seq dataset.

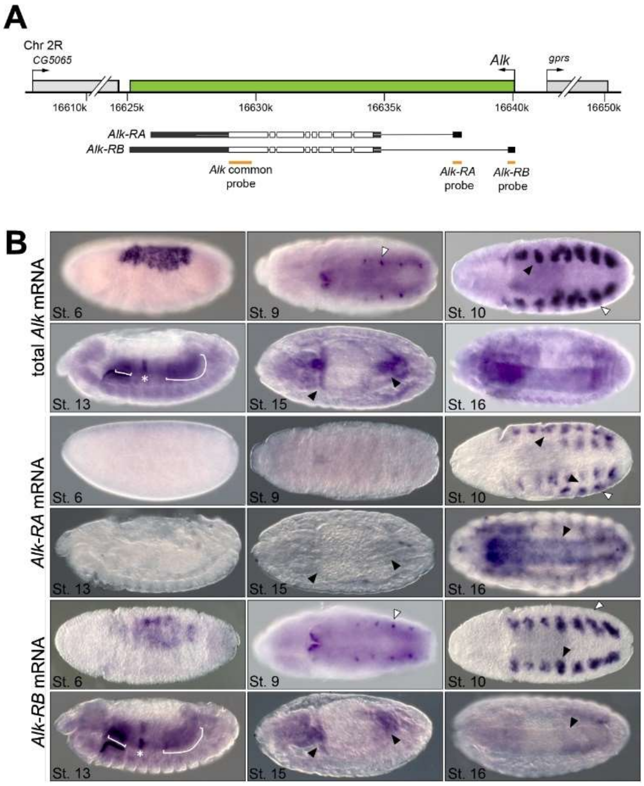

Fig. S5. Generation and characterization of the *Alk*$^{\Delta RA}$ mutant.

A Schematic outlining the genomic organization of the *Alk* locus (green) and including the neighboring genes *CG5065* and *gprs* (light gray). Intron-exon structure of *both Alk-RA* and *Alk-RB* transcripts is shown below (*Alk* open reading frame in white). Probes employed for *in situ* detection of common *Alk* mRNA, or *Alk-RA/Alk-RB* mRNA transcript specific expressions are indicated in orange.

B *In situ* showing expression of the common *Alk*, *Alk-RA* and *Alk-RB* transcripts during embryogenesis. Total *Alk* mRNA expression in wild type embryos. *Alk* transcripts are observed in the amnioserosa (stage 6), the trunk visceral mesoderm (VM) (stage 10, *closed arrowhead*) and the epidermis (stages 10, *open arrowhead*). *Alk* transcripts are present in the visceral muscle, particularly in PS7 (stage 13, *asterisk*) and PS3 (stage 15, *closed arrowhead*). Transcription of *Alk* is also observed in the developing CNS (stage

Disease Models & Mechanisms • Supplementary information

16). Expression of *Alk-RA* mRNA is observed in the epidermis in close proximity to the trunk VM (stage 10, *open arrowheads*) as well as strongly in the CNS (stage 16). *Alk-RB* mRNA is expressed in the amnioserosa (stage 6), as well as in the trunk VM (stage 10, *closed arrowhead*). The *Alk-RB* transcript is also observed at later stages in the VM, in PS7 (stage 13, *asterisk*) and PS3 (stage 15, *closed arrowhead*). Expression of the *Alk-RB* transcript was not detected in the embryonic CNS (stage 16, *closed arrowhead*).

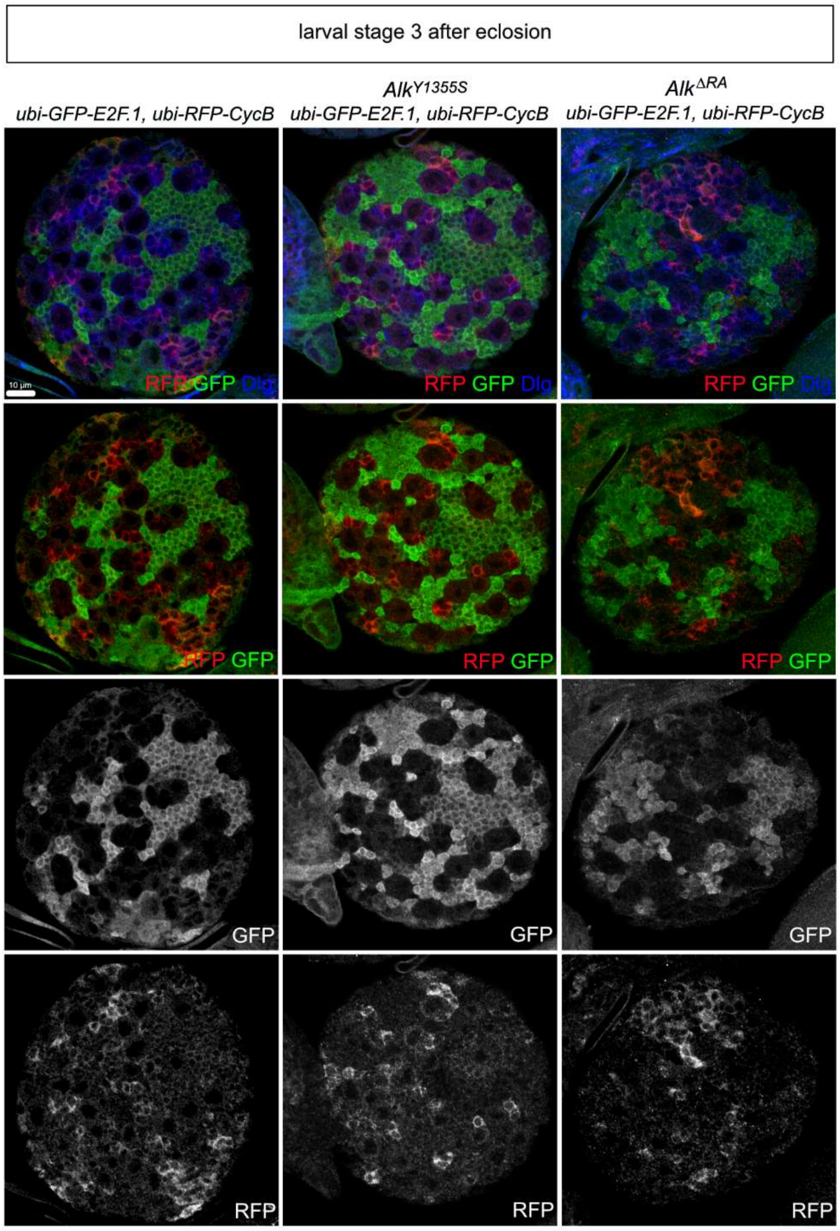

Fig. S6. FUCCI system employed in control, *Alk*^{Y1355S} and *Alk*$^{\Delta RA}$ at L3 stage after eclosion. Fly-FUCCI reporters *ubi-GFP::E2f1 (green) and ubi-mRFP::CycB* (red) denote G0/1- and S-phase cells, respectively. No gross differences in proliferation can be observed. Dlg2 shown in blue. Scale bar = 5 µm. n > 10.

Disease Models & Mechanisms • Supplementary information

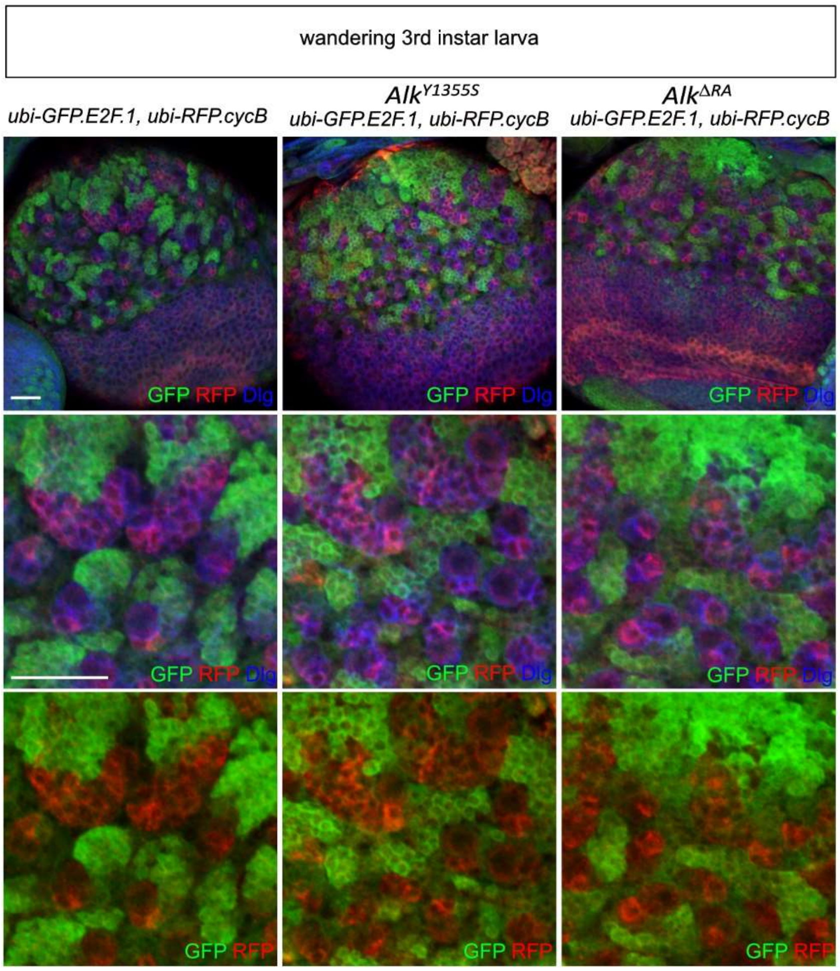

Fig. S7. FUCCI system employed in *control*, homozygous *Alk*^{Y1355S} and *Alk*$^{\Delta RA}$ in wandering third instar larvae. Fly-FUCCI reporters *ubi-GFP::E2f1 (green) and ubi-mRFP::CycB* (red) denote G0/1- and S-phase cells, respectively. No differences in proliferation in wandering third instar larvae can be observed. Dlg2 shown in blue. Scale bar = 20 µm. n > 10.

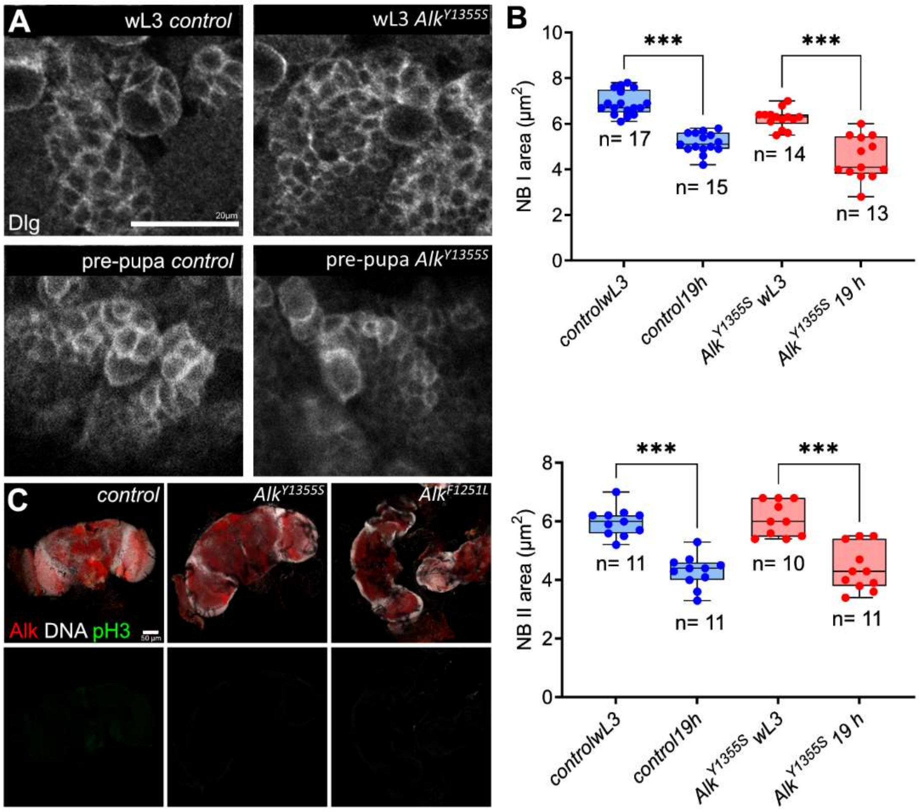

Fig. S8. Analysis of proliferation and NB quiescence in control and homozygous *Alk*^{Y1355S} adult brains.

A-B Type I and II NBs undergo shrinkage from wL3 until 19 h in *in vitro* brain culture. One-way ANOVA $p<0.001$, n = number neuroblasts analyzed in > 10 brains. Scale bar = 20 µm, **C.** No proliferation (pH3) can be detected in adult fly brains in either wildtype, homozygous *Alk*^{Y1355S} or homozygous *Alk*^{F1251L}. Scale bar = 50 µm.

Disease Models & Mechanisms • Supplementary information

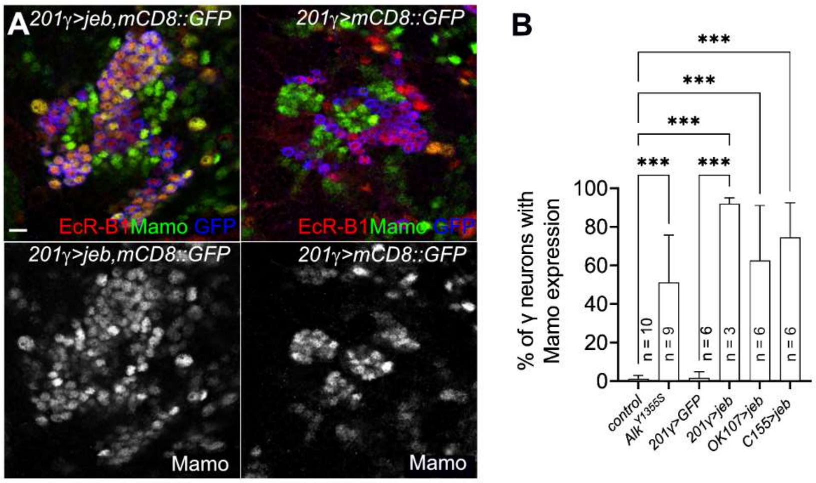

Fig. S9. Overexpression of Jeb using *201γ*-Gal4 and quantification of Mamo expressing cells in in the mushroom body lineage. A Ectopic Mamo expression is observed in wL3 brains in γ neurons after Jeb overexpression (*201γ-Gal4/+>jeb/+*). No Mamo is expressed in the γ lineage of controls (*201γ-Gal4/+>GFP/+*). Scale bar = 20 µm. B Quantification of Mamo expressing γneurons in different genotypes. One-way-ANOVA, ***p<0.001, n = number of analyzed brain lobes.

Disease Models & Mechanisms • Supplementary information

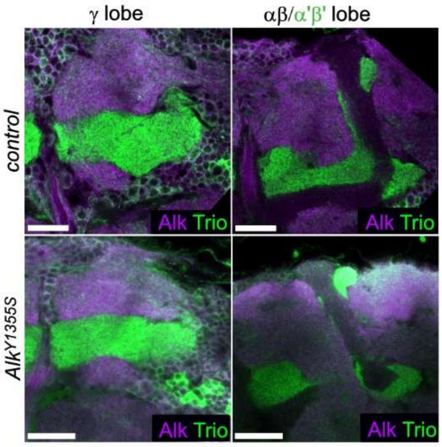

Fig. S10. Perturbed neuronal differentiation in *Alk*^{Y1355S} is maintained to adulthood.

Trio and Alk expression in MBs of homozygous *Alk*^{Y1355S} and control. Scale bar = 20 μm. n > 10 brains were analyzed.

Disease Models & Mechanisms • Supplementary information

CACGCGATAGTCTGCAATTGGTCAATGCTCTGGGCAAAGGTGCTTTTGGAGAGGTATATATGGCATTATATCGCCAT
CGAGACGGAGATGCTGTGGAAATGGGCGTGGCAGTGAAAACCTTGCGGGAAGATCCCAAGCGGGAAAAGGAGGAGGA
TTTCCTGAAGGAGGCGGCCATAATGGCCAAGTTCAATCACCCGAATATGGTCCATTTAATAGGAGTCTGCTTTGACC
GTCAGCCGTATTATATTGTCCTGGAACTTCTTGCTGGCGGGGATCTACAGAAGTTTTTGCGCGAAAACAGGAATACT
CCAGAGCGTCCCTCCCTCTTAACCATGAAGGATCTGCTTTTCTGCGCCCTCGACGTGGCCAAAGGATGTCGCTACAT
GGAGAGTAAACGCTTCATCCATCGGGATATCGCCGCCAGGAACTGTCTGCTGAGCAGTAAGGGTCCTGGTCGCGTTG
TGAAAA

Tt GCt GAc TTc GGt ATG TCa CGG GAT ATCTcC CGA TCa GAt TAt TAc CGt AAa GGt GG

CAAGGCGATGCTACCCATCAAATGGATGCCGCCGGAAGCCTTCCTCGATGGCATATTCACCTCGAAAACAGATGTCT
GGTCCTTTGGCATTCTCCTGTGGGAAGTCTTCAGTCTGGGCCGTTCACCATATCCCGGCCAGCATAACACCCAGGTA
ATGGAGCTGGTTGTGCGTGGCGGTCGACTGGGATCGCCCACCGAGTGTCCCGTCTCCATATACAAGGTGATGGCCGA
CTGCTGGAATCCCACGCCCGAGGATCGGCCCACATTTATTACGTTGCTGGAGCATTTGACCGCCTGCACCCAGGATG
CCAGCATAATGAACGCTCCTCTGCCCAATATTCTGGGACCCACAGCCTCCGAACGGGACGACACAGTCATCCGGCCG
CCCAATGGCGAGGAGTTCTGTTTGGCGGTGCCGGATTATCTGGTACCTTTGCCACCCGGCGGCAGCAACAATCCATC
GATGGCCAG

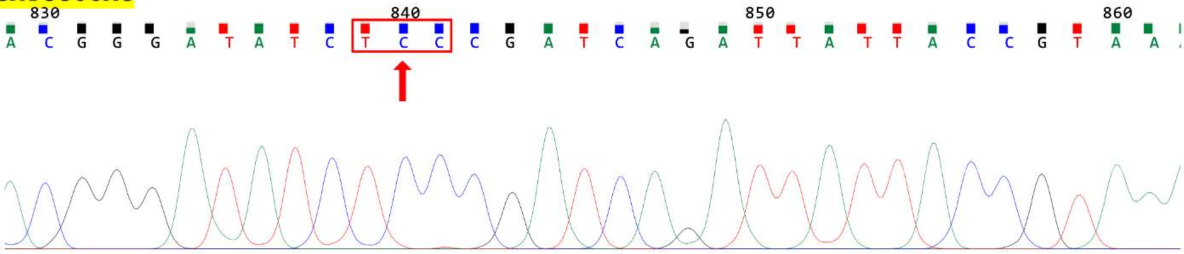

gRNA Y1355S nr.1 TCGCCGATTTTGGCATGTCCCGG

gRNA 2 Y1355S nr.2 TCGGACTACTATCGCAAGGGAGG

Fig. S11. *Alk*^{Y1355S} donor construct sequence.

Donor construct design: pink and green regions in donor sequence containing silent mutations to prevent the donor construct from destruction during homology induced repair (HDR). Homology arms in yellow. The homology arms are integrated into the endogenous locus during HDR**.** Chromatogram file from sequenced *Alk*^{Y1355S} mutant showing the desired mutation (red arrow), resulting in a change of amino acid from tyrosine to serine (serine, red box). Guide RNA in pink and green.

Disease Models & Mechanisms • Supplementary information

GAGCCCTTGAATAAGAAGACAGCTGGTCCAGGTGGTGGATGGAGAGCCAAAGAGGACCAGGCCCTGAG
TCCCACCTATGGAGCTGCCCTTCTCCAGGGCGGTCGAGGTGGACACTCCTGTTATGTGGAACTTGCGG
ACAATGGAACTTCTGTTCACAGACATGGCCAAGGTGGATTTGGTGGTGGCGGTGGTGGCTGCAATACT
GGAGGAGGCGGTGGCGGCTACGCTGGCGGAGATGTCTACCTAACCGAGTCGAATGGCGAGGGCGGAAG
TTCGTACATCAGCCCCAGTCGAAGTCTTCGGGAAATTAGTGAAATACATGCCGGCGCTAGCAGTGGAC
CAGGTGCCATCATCATTATTCCGGCAATCGAGGGCTGTGGATGCGACTATAGGTGCGTGGCCTTGGAT
GAGTTCCGATCGAAGGTGCGGTGCATCTGCCCAGATGGCTGGAGCTTAAAACGGGACAATCACACCGC
CTGTGAGATCCGCGAGGAGGCGGGCAAGTCCTCCTTCCAGTATCTGGTTTCCATCCTAATGATCTCGC
TTGCCGTGCTCTTCATCTGCATAGCTGCGCTCATCTTTATGCTATgtaagtattcaagaaattctgtt
tgtatgaatggaaatgatattctctttttacattagATAATCGCTATCAACGGAAGAAACAATCGAAG
AAGCGCCACAAGATGCTCGTGGAGCAGGATCTTCAACTTACCCGTTTGCGCAACAATATCGACGACTC
CAATCTGAATAACTTTAACCCCAACTATGGATGTGATGGTATTCTCAATGGTCACATTGATGTCAATA
GCTTGCCCCAGGTGGCACGCGATAGTCTGCAATTGGTCAAgtgagtaacttaatctaatatataacct
tatgtgatctacaaatgtgattcaatcgttttcagTGCTCTGGGCAAAGGTGCTTTTGGAGAGGTATA
TATGGCATTATATCGCCATCGAGACGGAGATGCTGTGGAAATGGGCGTGGCAGTGAAAACCTTGCGGG
AAGATCCCAAGCGGGAAAAGGAGGAGGATTT

CCTa AAa GAa GCa GCa ATc ATG GCa AAg **c**Tt AAc CAt CCa AAc ATG G

TCCATTTAATAGGAGTCTGCTTTGACCGTCAGCCGTATTATATTGTCCTGGAACTTCTTGCTGGCGGG
GATCTACAGAAGTTTTTGCGCGAAAACAGGAATACTCCAGAGCGTCCCTCCCTCTTAACCATGAAGGA
TCTGCTTTTCTGCGCCCTCGACGTGGCCAAAGGATGTCGCTACATGGAGAGTAAACGCTTCATCCATC
GGGATATCGCCGCCAGGAACTGTCTGCTGAGCAGTAAGGGTCCTGGTCGCGTTGTGAAAATCGCCGAT
TTTGGCATGTCCCGGGATATCTACCGATCGGACTACTATCGCAAGGGAGGCAAGGCGATGCTACCCAT
CAAATGGATGCCGCCGGAAGCCTTCCTCGATGGCATATTCACCTCGAAAACAGATGTCTGGTCCTTTG
GCATTCTCCTGTGGGAAGTCTTCAGTCTGGGCCGTTCACCATATCCCGGCCAGCATAACACCCAGGTA
ATGGAGCTGGTTGTGCGTGGCGGTCGACTGGGATCGCCCACCGAGTGTCCCGTCTCCATATACAAGGT
GATGGCCGACTGCTGGAATCCCACGCCCGAGGATCGGCCCACATTTATTACGTTGCTGGAGCATTTGA
CCGCCTGCACCCAGGATGCCAGCATAATGAACGCTCCTCTGCCCAATATTCTGGGACCCACAGCCTCC
GAACGGGACGACACAGTCATCCGGCCGCCCAATGGCGAGGAGTTCTGTTTGGCGGTGCCGGATTATCT
GGTACCTTTGCCACCCGGCGGCAGCAACAATCCATCGATGGCCAGTGGTTCGGGCTACGTGCCAGAAT
TGCAACGCCAGCAGATGAGCTCCTGCACTCCGCCGGCGGTCACATCGCCCGCCGCACCACATCCAAGG
CCGGTGGAGAATATCGCA

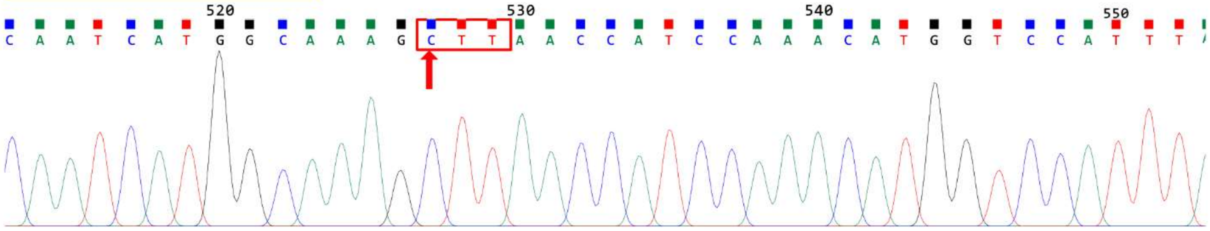

gRNA F1251L nr.1 CCTGAAGG AGGCGGCCATAA TGG

gRNA F1251L nr.2 CAAGTTCA ATCACCCGAATA TGG

Fig. S12. ***Alk*^{F1251L} mutant donor construct sequence.**

Donor construct design: pink and green regions containing silent mutations in donor sequence to prevent the donor construct from destruction during homology induced repair

Disease Models & Mechanisms • Supplementary information

(HDR). In red the changed codon. The homology arms are integrated into the endogenous locus during HDR. Chromatogram file from sequenced *Alk*^{F1251L} mutant showing the desired mutation (red arrow) resulting in a change of amino acid from phenylalanine to leucine (serine, red box). Guide RNA in pink and green.

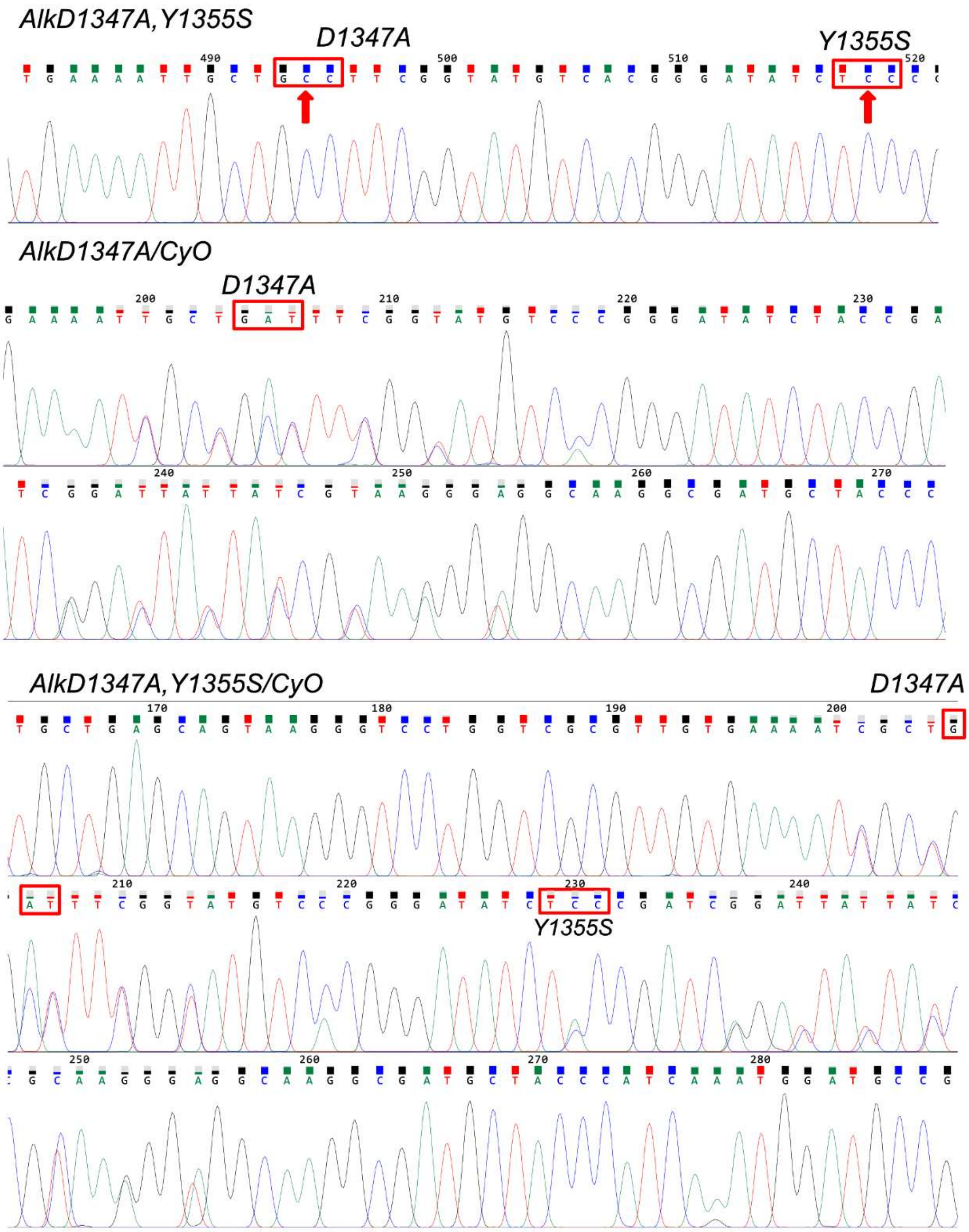

Fig. S13. Sequence confirmation of the *Alk*$^{D1347A,\ Y1355S}$ double mutant

Chromatograms showing sequence of the *Alk*$^{D1347A,\ Y1355S}$ double and homozygous mutant . Red boxes highlight modified codons, red arrows indicate mutated nucleotide.

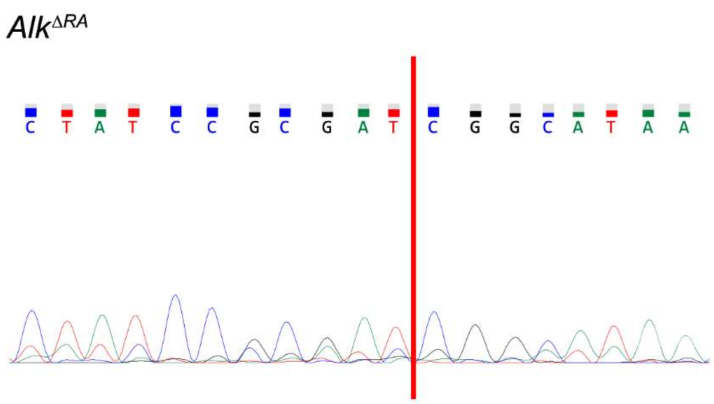

Fig. S14. Chromatogram showing sequence of the *Alk*$^{\Delta RA}$ mutant. Red line indicates breakpoint region of CRISPR/Cas9 induced deletion.

Disease Models & Mechanisms • Supplementary information
